# Extraction, analysis and presentation of results in a scoping review in Brazilian nursing: scoping review

**DOI:** 10.1590/1518-8345.7832.4786

**Published:** 2026-03-16

**Authors:** Isabelle Cristinne Pinto Costa, Patricia Treviso, Lucélia Terra Chini, Murilo César do Nascimento, Andreia Cristina Barbosa Costa, Patrícia Scotini Freitas, Karina Dal Sasso Mendes

**Affiliations:** 1 Universidade Federal de Alfenas, Escola de Enfermagem, Alfenas, MG, Brazil.; 2 Universidade Federal do Rio Grande do Sul, Escola de Enfermagem, Porto Alegre, RS, Brazil.; 3 Universidade de São Paulo, Escola de Enfermagem de Ribeirão Preto, PAHO/WHO Collaborating Centre for Nursing Research Development, Ribeirão Preto, SP, Brazil.

**Keywords:** Nursing, Data Analysis, Brazil, Evidence-Based Practice, Evidence-Based Nursing, Scoping Review.

## Abstract

**Objective:**

to map the literature on extraction, analysis, and presentation of results in scoping reviews in Brazilian nursing.

**Method:**

scoping review according to the Joanna Briggs Institute guidelines, including publications, in the following sources: PubMed, Latin American and Caribbean Literature in Health Sciences, CINAHL, Embase, Scopus, Web of Science Core Collection, Cochrane Database of Systematic Reviews, and Google Scholar. The search was structured in three stages and conducted by two independent reviewers.

**Results:**

one hundred and forty-three studies were included; the annual number increased from one (2013) to 41 (2023). In the title, 90.2% stated “scoping review”; 74.1% followed the JBI framework, but only 60.8% cited PRISMA-ScR, and 39.2% registered a protocol. Extraction remained largely manual: 82.5% used adapted JBI forms, and only 11.9% used support software. Descriptive summaries and thematic categorization predominated. Presentation favored charts and tables (96%).

**Conclusion:**

Nursing scoping reviews in Brazil use standardized procedures, but lack greater adherence to PRISMA-ScR, prior registration, and digital technologies. Training in international guidelines, AI, and interactive visualizations are priorities to increase transparency, reproducibility, and clinical impact.

## Highlights

**(1)** Mapping extraction and analysis practices in nursing scoping reviews. **(2)** Growth in scoping reviews over the last decade, with 143 studies included. **(3)** Methodological guidelines followed; technologies and AI still underexplored. **(4)** Low adoption of PRISMA-ScR indicates the need for greater training. **(5)** Scoping reviews strengthen evidence for nursing practice in Brazil.

## Introduction

Evidence synthesis encompasses various types of reviews, with scoping reviews being an increasingly adopted approach to summarize evidence in different fields. This type of review gained popularity after 2012^1^ ^-^ ^2^.

The first methodological guide for scoping reviews was published by Arksey and O’Malley in 2005, offering an initial framework based on their observations and reflections on existing scoping studies. In 2010, Levac and colleagues proposed refinements to this framework^3^. Subsequently, in 2014, the Joanna Briggs Institute (JBI) and its network of collaborators conducted an extensive literature review, resulting in published recommendations on the development of scoping reviews^4^ ^-^ ^6^. These guidelines have been refined in successive methodological guides to support researchers, developed by scoping review experts and published in 2017, 2020, and, most recently, in 2024^7^ ^-^ ^9^.

According to the JBI, a scoping review is an evidence synthesis aimed at systematically and comprehensively mapping the scope and types of available studies on a topic, concept, or clinical question, bringing together primary research, reviews, and other relevant sources^2^.

To conduct a scoping review rigorously, it is essential to develop a preliminary protocol detailing the objective, research questions, and eligibility criteria, as well as adopting a broad search strategy followed by thorough screening. The participation of at least two independent reviewers and adherence to specific guidelines are recommended - especially the Preferred Reporting Items for Systematic Reviews and Meta-Analyses for Scoping Reviews (PRISMA-ScR) reporting guide, which standardizes reporting and enhances process transparency^1^ ^,^ ^10^.

The data extraction and analysis stages of scoping reviews began to be addressed in greater detail after the publication, in 2023, of specific JBI recommendations that standardize forms, coding strategies, and synthesis methods^11^.

In Brazil, the social distancing imposed by COVID-19 accelerated this agenda: given the volume of emerging studies, many research groups turned to scoping reviews to quickly organize evidence and inform clinical and public policy decisions. The flexibility of the method - capable of encompassing different study designs and information sources - proved particularly useful in the pandemic context.

As the methodology advances, it becomes essential for nurses to adopt the most recent guidelines, especially when the results are intended to guide new research or inform care practices^12^.

In March 2023, an exploratory search was conducted in the Open Science Framework (OSF), PubMed, and JBI Evidence Synthesis to identify scoping reviews - published or in progress - on the extraction, analysis, and presentation of results in scoping reviews in nursing. No reviews with this scope were found. Only (i) one methodological article discussing guidelines for scoping reviews in Nursing and Midwifery^12^ and (ii) a systematic review published in 2022 describing the methodological characteristics of scoping reviews in nursing journals were retrieved, highlighting inconsistencies in the data analysis and presentation stages and recommending the use of the PAGER framework to standardize the reporting of findings^13^ ^-^ ^14^. These findings highlight the gap that the present study aims to fill.

The lack of syntheses that specifically address the procedures for extracting, analyzing, and presenting results in scoping reviews conducted by nursing researchers in Brazil highlights a methodological gap. Therefore, this scoping review aims to map the literature on extracting, analyzing, and presenting results in scoping reviews in the context of Brazilian nursing.

## Method

### Protocol and registration

This study was conducted according to the methodology established by the Joanna Briggs Institute (JBI) for scoping reviews. The steps followed included: defining and aligning the objective and research question; developing inclusion criteria aligned with the objective; describing the approach to searching, selecting, extracting, and presenting the evidence; and executing these steps with subsequent analysis and summary of the evidence, highlighting the conclusions and implications of the findings^9^. The methodology was chosen because it allows for a comprehensive mapping of the evidence, appropriate for the objectives of this study. The protocol was registered in the OSF repository on May 2^nd^, 2023^15^, and the PRISMA-ScR guideline was used to report the results^1^.

### Eligibility criteria

This review addressed the following research question, structured by the mnemonic Population, Concept, and Context (PCC): How have scoping reviews been conducted regarding the extraction, analysis, and presentation of results in the Brazilian nursing context?

The study population included publications that used scoping reviews as the primary method. Studies that employed different methodologies were excluded, even if scoping reviews were a step in the research process. Regarding the concept, studies that described the extraction, analysis, and presentation of results in scoping reviews were considered. The focus was on how these processes were described and executed by the researchers. Regarding the context, the review covered studies conducted within the Brazilian nursing context, with at least one nurse as an author. Studies from other health fields or those involving Latin American countries other than Brazil were excluded.

Studies published in any language were included. The review covered publications from 2005 to February 2024, delimiting the emergence of the first methodological guidelines^16^.

### Information sources

After protocol validation, searches were conducted across several data sources, selected for their relevance to health and nursing. The following sources were used: Latin American and Caribbean Literature in Health Sciences (LILACS), maintained by the Virtual Health Library (VHL); PubMed, the biomedical literature repository of the United States National Library of Medicine (NLM); The Cumulative Index to Nursing and Allied Health Literature (CINAHL), an essential source for nursing literature, published by EBSCO Information Services; Embase, aggregated by Elsevier; Scopus, one of the largest multidisciplinary databases, also aggregated by Elsevier; the Cochrane Database of Systematic Reviews, published by the Cochrane Collaboration and accessible via the Wiley Online Library; and the Web of Science Core Collection (WOSCC), maintained by Clarivate Analytics.

In order to identify gray literature, searches were conducted on Google Scholar. 

### Literature search

The search strategy was developed and implemented in three distinct stages:

- Preliminary search: This involved conducting a preliminary search in the CINAHL and LILACS databases to assess the feasibility of the proposed review and to validate and align the terms to be used in the search strategy. Controlled descriptors and synonyms from the Thesaurus of Health Sciences Descriptors (DeCS/MeSH), CINAHL Titles, and Emtree terms were identified. Furthermore, keywords contained in the titles and abstracts of the relevant articles analyzed were analyzed.

- Term refinement and strategy development: Based on the information collected in the preliminary stage, the second stage consisted of the final search strategy for PubMed. The strategy included all relevant keywords and indexed terms contained in the Medical Subject Headings (MeSH), combined with the terms identified in the previous stage. It was developed with the input of the study team and reviewed by a specialized librarian to ensure its accuracy and comprehensiveness.

- Adaptation to other information sources: in the third stage, the search strategy developed for PubMed was adapted to the other selected information sources. This involved adjusting the commands to adapt to the specific syntax of each information source and using Boolean operators AND and OR to combine controlled and uncontrolled terms, optimizing the retrieval of relevant studies in each source.

It was decided not to manually search the reference lists of the included studies, as the final sample was considered sufficiently broad and representative, allowing for a complete and detailed analysis of the scientific literature on the topic. The search was implemented on February 20^th^, 2024. The search strategies were adapted to the information sources, as per the example of the search performed in PubMed, described below: (“Scoping review”[Title/Abstract] OR “Literature Mapping”[Title/Abstract] OR “Mapping Review”[Title/Abstract] OR “Mapping of Research”[Title/Abstract] OR “Scoping Report”[Title/Abstract] OR “Scoping Study”[Title/Abstract] OR “Preliminary review”[Title/Abstract] OR “Scope review”[Title/Abstract] OR “Comprehensive review”[Title/Abstract] OR “Mapping study”[Title/Abstract] OR “Preliminary investigation”[Title/Abstract] OR “Evidence mapping”[Title/Abstract]) AND (“Nursing”[MeSH Terms] OR “Nursing”[Title/Abstract] OR “Nursings”[Title/Abstract] OR “Nurses”[MeSH Terms] OR “Nurses”[Title/Abstract] OR “Nurse”[Title/Abstract] OR “Registered Nurse”[Title/Abstract]) AND (“Brazil”[MeSH Terms] OR “Brazil”[Title/Abstract] OR “Brazil”[All Fields] OR “Brazilian”[All Fields] OR “Brazilian”[All Fields]).

### Study selection

After searching the information sources, all identified records were grouped and uploaded to EndNote 20 software, where duplicates were removed^17^. The records were then exported to the online application Rayyan, used for the study selection stage^18^. Two independent reviewers, working individually, analyzed the titles and abstracts to assess compliance with the previously established selection criteria.

The full texts of potentially relevant studies were then assessed in detail by two independent reviewers, also individually, according to the inclusion criteria. The reasons for exclusion of any studies whose full text did not meet the inclusion criteria were recorded and reported in the final scoping review. Any disagreements between the reviewers, at any stage of the study selection process, were resolved through discussion or by the intervention of a third reviewer.

### Data extraction and analysis

Data were extracted from the included studies by two independent reviewers to minimize errors and biases. A data extraction script proposed by the JBI^14^ was used, which was adapted by the reviewers and developed in Microsoft Excel.

The extracted data included detailed information on the population, concept, and context^8^. The extraction form was structured to capture specific information, such as author identification, title, year, language, and journal of publication, as well as the first author’s institution and the journal’s country of origin. Data were also extracted on the study objective, the clarity in identifying the scoping review as the methodology used, the topic addressed, and the methodological framework followed. Additionally, the form verified whether the methodological steps were described and whether the study adhered to the PRISMA-ScR guide, including the use of a flowchart and documentation of the review protocol. We also assessed whether the search strategy was documented in detail, with emphasis on the use of the PCC mnemonic, definition of the research question, eligibility criteria, and application of filters or limiters^8^ ^,^ ^14^.

Data extraction covered both bibliometric information and PCC elements, considering the use of software such as EndNote and Rayyan to aid in study selection. Data analysis focused on whether the search strategy was described in terms of descriptive and quantitative methods, using graphs and tables. The number of studies included, any limitations identified, and any knowledge gaps highlighted by the authors were also reported. A data extraction guidance form was developed to accompany the main form, detailing each item to be extracted and shared with the reviewers.

During data extraction, a pilot test was conducted, in which each reviewer independently completed data extraction from at least three studies. During the pilot test, reviewers evaluated the form by answering questions about missing items, redundancy, clarity, and time required for data extraction. These reflections helped adjust the timing and structure of the extraction^11^.

The scoping review team held regular meetings to facilitate communication throughout the data extraction process, which was an iterative process to ensure it met the review objectives.

While not required, contact with the authors of the included studies to request missing or additional data was planned.

### Summary or summary of results

A mixed-method approach was used to analyze the data for this scoping review, combining qualitative and quantitative methods. Initially, open coding was performed, allowing researchers to identify relevant themes and concepts directly from the raw data in an impartial manner. From this coding, preliminary categories were developed that reflected the main areas of interest of the study.

Due to the large number of studies included in the sample, it was necessary to use artificial intelligence (AI), such as ChatGPT 4.0, to aid in the categorization process. With the help of AI, it was possible to optimize the identification of patterns and trends, facilitating the grouping and refinement of categories with greater precision. The content generated with ChatGPT 4.0 was subjected to careful validation by two independent reviewers with experience conducting scoping reviews and familiarity with qualitative analysis methods.

The categorization process followed the principles of basic qualitative content analysis, as recommended in the updated JBI guidelines for scoping reviews^11^, including the steps of open coding, category development, and cross-review.

The prompt used to assist with categorization was the following: “Act as a scoping review expert. Below, a table with titles and abstracts of included studies will be presented. Based on the content, identify emerging themes and group the studies into thematic categories, justifying each grouping based on conceptual similarity”.

The data were not directly uploaded to the AI, but rather organized into a table with study information (authors, title, abstract, and keywords) and manually entered into the secure environment of the ChatGPT 4.0 tool.

The generated content was used to support the initial coding, not as a final categorization. Final validation of the categories and subcategories was performed by the research team, with decisions recorded in minutes and traceability spreadsheets. This process aimed to make the analysis stage more efficient and transparent, without compromising methodological rigor. The derived categories were subsequently discussed in team meetings, ensuring the principle of data triangulation. The use of AI was therefore complementary, assisted, and supervised, not a substitute for human analysis.

Concurrently, a simple quantitative analysis was conducted, in which frequencies and percentages were calculated to describe the distribution of data in relation to key variables, such as themes covered and demographic characteristics of the studies. Furthermore, with the help of AI, it was easier to create figures, which were generated from CANVA, making the visualization of the results clearer and more accessible. These visual representations facilitated the interpretation of the results, providing a comprehensive and clear view of the evidence collected.

The results of the study selection process were synthesized in a PRISMA-ScR flowchart^1^. 

## Results

**Figure 1:**
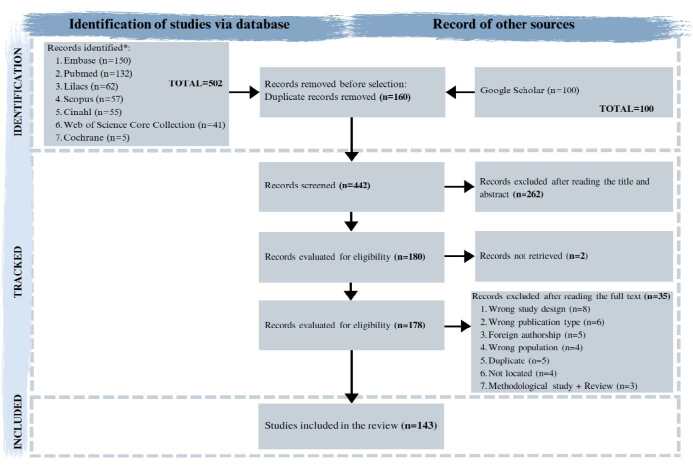
Flowchart for identification, selection, and inclusion of scoping review studies according to PRISMA-ScR^1^ guidelines. Alfenas, MG, Brazil, 2024

A total of 602 records were identified in the PubMed (n=132), LILACS (n=62), CINAHL (n=55), Embase (n=150), Scopus (n=57), WOSCC (n=41), Cochrane (n=5), and Google Scholar (n=100) databases. After exporting to EndNote, 160 duplicate records were removed, resulting in 442 studies that proceeded to the screening process in Rayyan. In the first phase of screening, performed by title and abstract, 262 records were excluded, which led to 180 studies being read in full, of which two were not recovered. At this stage, 35 studies were also excluded after full reading. Thus, 143 studies were included for data extraction and analysis, as illustrated in Figure 1.

The distribution of studies by year of publication revealed significant trends in the evolution of scientific production related to scoping reviews. The highest peak in publications occurred in 2023 (41; 28.7%), followed by 2021 (36; 25.2%) and 2022 (30; 21.0%). There was also significant production in 2020 (14; 9.8%) and 2019 (9; 6.3%). Previous years presented a smaller number of publications, such as 2018 (4; 2.8%), 2017 (2; 1.4%), 2016 (1; 0.7%), and 2015 (3; 2.1%). As of the date of the search in the information sources, three (2.1%) studies were identified for the year 2024.

Analysis of the languages of publication of the 143 studies revealed significant linguistic diversity in the scientific production. English stood out as the most frequently used language, with 38 (26.6%) studies published exclusively in this language. The combination of “English, Portuguese, and Spanish” appeared most frequently, with 46 (32.2%) publications. The combined use of “Portuguese and English” was also significant, with 39 (27.3%) studies. Portuguese was the exclusive language of 18 (12.6%) studies, while Spanish was exclusive to only one (0.7%) study, as was the combination of “Portuguese and Spanish,” which also appeared in one (0.7%) publication.

**Figure 2:**
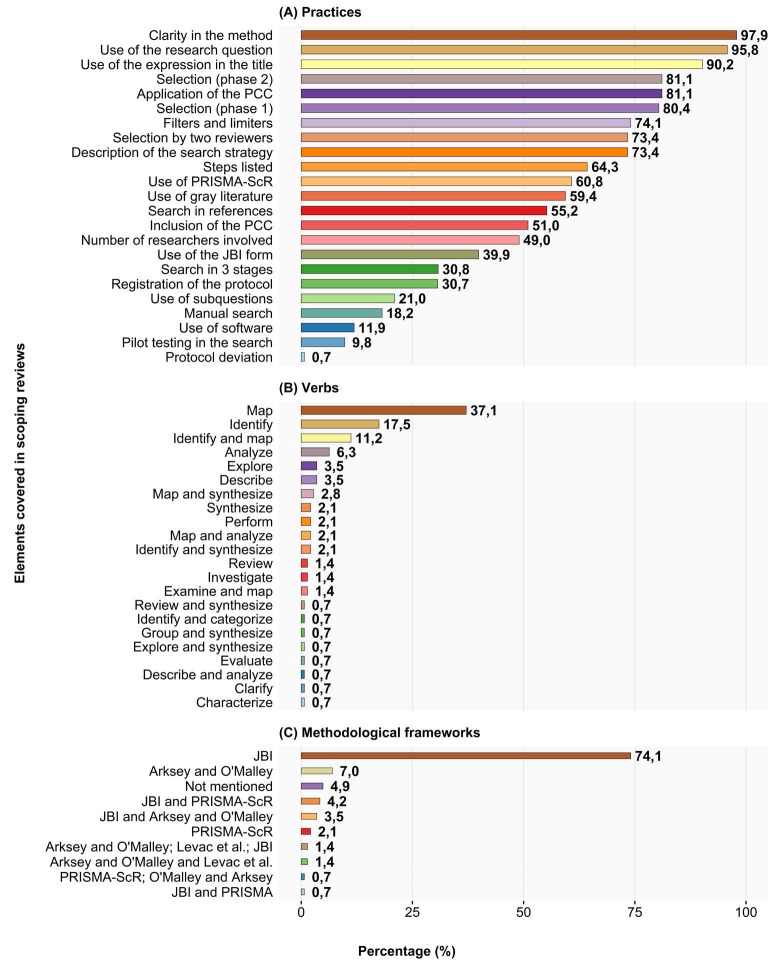
Methodological overview of scoping reviews in Brazilian nursing. Alfenas, MG, Brazil, 2024

Figure 2 illustrates the methodological procedures adopted in scoping reviews conducted by nurses in Brazil, highlighting the use of several important practices and trends. Most studies (140; 97.9%) clearly identified their scoping review in their methods, and 129 (90.2%) made this identification directly in the study title. The verb “map” was the most frequently used, appearing in 53 objectives, representing 37.1% of the total. The most frequently used theoretical framework was the JBI, used in 106 (74.1%) studies, followed by Arksey and O’Malley, with 10 (7.0%) studies. Furthermore, a combination of the JBI with the Arksey and O’Malley guidelines was used in five (3.5%) studies. Most studies (87; 60.8%) used PRISMA-ScR to describe their research. A significant portion of the studies (92; 64.3%) adopted a structured approach, clearly defining the specific steps of the scoping review. However, only 56 (39.2%) studies mentioned protocol registration. Among the studies, 116 (81.1%) used the PCC mnemonic to structure their scoping reviews. The research question was mentioned in 137 (95.8%) studies, while only 30 (21.0%) included research subquestions.

The vast majority of studies (106; 74.1%) mentioned the use of filters and limiters in their search strategies. The most commonly used filter was a combination of time frame and language, applied in 32 (30.2%) studies. Next, a filter by language alone was mentioned in 28 (26.4%) studies. Other filters were reported, including: time frame alone in 9 (8.5%) studies, a combination of filters by language and source types in 5 (4.7%) studies, and a filter by available documents in full combined with language, present in 6 (5.7%) studies. Less frequent filters included a combination of language, sources, and available documents in full in 1 (0.9%) study, as well as combinations involving time frame and sources, or time frame and full documents, each mentioned in 1 (0.9%) study. The filter involving only source types was used in 3 (2.8%) studies. Furthermore, 6 (5.7%) studies did not detail the specifics of the filters used.

Only 14 (9.8%) studies reported conducting a pilot test of the search strategy. The search strategy was developed in three stages in 44 (30.8%) studies. More than half of the studies (85; 59.4%) used gray literature as an information source, and manual searches of specific sources were mentioned in 26 (18.2%) studies. Searching the references of the included studies was used in 79 (55.2%) studies. The search strategy was presented in the majority of studies (105; 73.4%). Study selection by title and abstract (Phase 1) and by full-text reading (Phase 2) was widely practiced, with 115 (80.4%) and 116 (81.1%) of the studies indicating these stages, respectively. Selection by two reviewers was mentioned in 105 (73.4%) of the studies. Regarding the distribution of studies selected and included in scoping reviews, categorized into four distinct intervals based on the number of studies, the low and moderate intervals (1-50 studies) dominated the distribution of scoping reviews, together representing 117 (81.9%) studies. Reviews involving a larger number of studies (51-100) were less common, accounting for 18 (12.5%) studies, while extreme reviews (more than 100 studies) were quite rare, accounting for only 5.6%.

**Figure 3:**
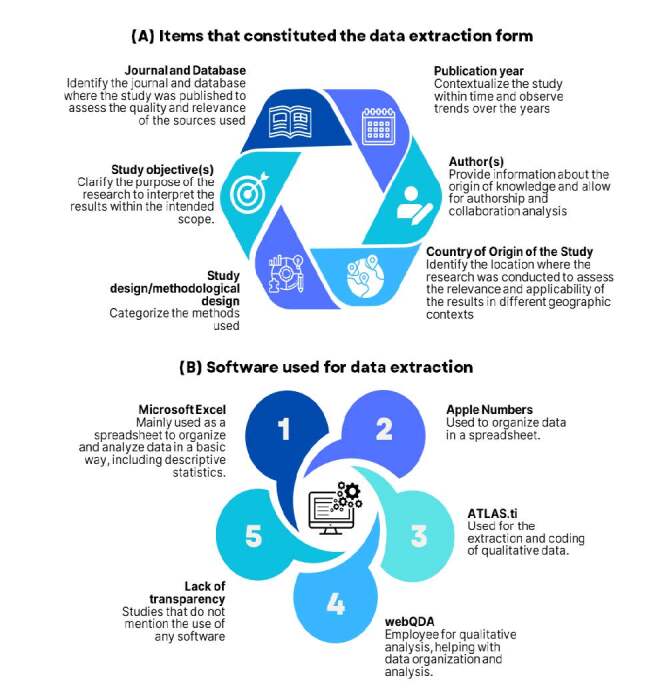
Items from the forms and software used in the data extraction process for scoping reviews in Brazilian nursing. Alfenas, MG, Brazil, 2024

Figure 3 addresses the data extraction process. First, it was observed that 57 (39.9%) studies adopted the JBI form, one of the most recognized standards for systematic data extraction. Regarding the inclusion of PCC elements in the extraction forms, 73 (51%) of the studies explicitly mentioned their application. Another relevant point was adherence to the planned protocols. Only one (0.7%) reported protocol deviations in data extraction. Regarding the use of technologies, only 17 (11.9%) studies explicitly mentioned the use of software for data extraction, such as Microsoft Excel, ATLAS.ti^®^, webQDA, and Apple Numbers. Regarding the number of researchers involved in data extraction, 70 (49%) studies described this information.

**Figure 4:**
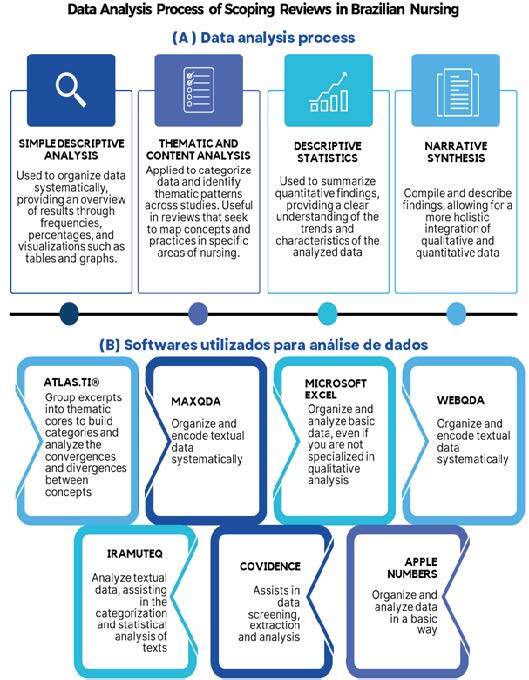
Data analysis process and software used in scoping reviews in Brazilian nursing. Alfenas, MG, Brazil, 2024

Figure 4 provides a comprehensive overview of the data analysis process, highlighting both the software used and the analysis methods employed. The main software programs used for data analysis included qualitative tools such as ATLAS.ti^®^, MAXQDA, webQDA, and IRaMuTeQ, as well as more general-purpose software such as Microsoft Excel and Apple Numbers. These programs played a key role in organizing, coding, and systematically analyzing the data. Regarding the methodological approaches adopted, simple descriptive analysis, as well as thematic and content analysis, were widely used to organize and categorize the data, respectively.

**Figure 5:**
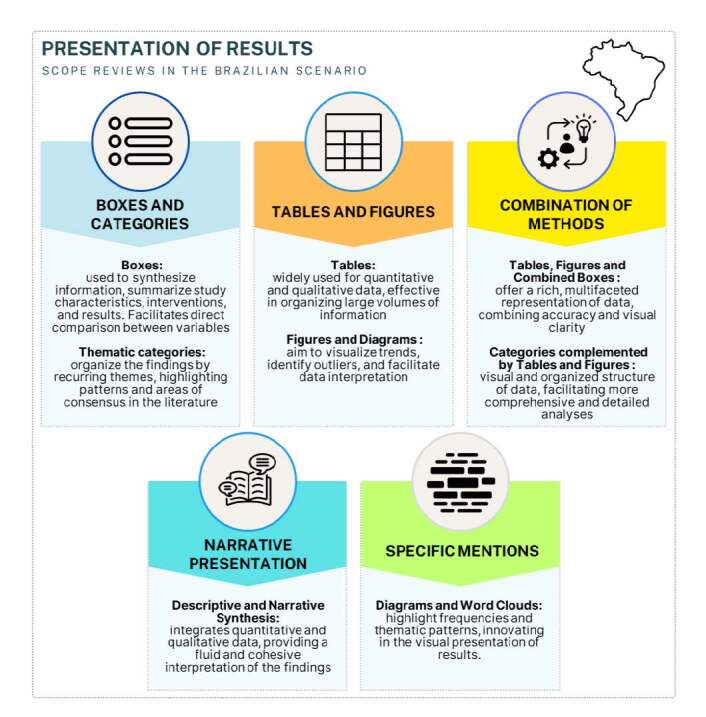
Forms of presenting results in scoping reviews in Brazilian nursing. Alfenas, MG, Brazil, 2024

Figure 5 highlights the main ways results were presented in scoping reviews in Brazil, with a predominance of the use of tables and thematic categories, which organized and synthesized complex information, facilitating comparisons and the identification of patterns. Tables and graphs were also widely used.

The scoping reviews examined revealed a significant diversity of topics, grouped into three main areas: clinical-care, population-based, and educational-political. In the clinical field, research focused on palliative care, mental health, patient safety, healthcare-associated infections, and nursing diagnostic processes.

Regarding emerging approaches, there was a growing emphasis on advanced nursing practices, digital technologies applied to training and care, and forensic nursing. At the population level, studies included vulnerable groups, such as the elderly, LGBTI+ individuals, Indigenous people, people deprived of liberty, migrants, and refugees. Research focused on specific clinical conditions - such as hypertensive disorders during pregnancy, cancer, Parkinson’s disease, and COVID-19 infections in multiple profiles - as well as topics such as therapeutic adherence in tuberculosis and pain management strategies - was also identified. In the formative and ethical-political domain, the topics addressed were active methodologies, clinical simulation, the use of digital games, learning styles, human rights in the care of people with mental disorders, and forensic preservation actions in contexts of violence. Furthermore, analyses emerged related to the management and formulation of public policies, such as mental health organizational models, professional oversight, hospital sustainability, and access to services in border regions. This thematic overview reaffirms the relevance of scoping reviews in consolidating nursing knowledge and guiding priorities for future research agendas.

## Discussion

This is the first mapping to quantify and interpret the methodological evolution of scoping reviews produced by Brazilian nurses. The temporal analysis revealed a significant growth in the number of scoping reviews published by Brazilian nurses over the past three years (2021-2023). This expansion appears to have been driven by responses to global crises, including the COVID-19 pandemic, as well as by the need for comprehensive approaches to complex health issues, characteristics inherent to scoping reviews^19^. As previously suggested in the literature, the flexibility of this approach in rapidly changing knowledge landscapes^15^ has consolidated the methodology in the Brazilian nursing field.

However, the quantitative advance was not accompanied by a proportional improvement in methodological quality. Although 90.2% of studies indicated in their title that they were scoping reviews and 74.1% adopted the JBI framework, only 60.8% used the PRISMA-ScR guide, and 39.2% mentioned protocol registration. In almost two-fifths of publications, therefore, there remains a significant risk of poor transparency and reproducibility, a scenario also described in international nursing journals^13^.

These findings reveal opportunities for improving researcher training, particularly regarding the importance of rigorous adherence to methodological guidelines, aiming to increase the robustness and transparency of reviews and maximize their impact on clinical, educational, and managerial practice^1^ ^,^ ^10^ ^,^ ^12^.

Although most studies described the steps of the scoping review, a significant portion omitted relevant details of the process, highlighting the need for greater clarity and systematization^8^ ^,^ ^13^. The use of the PCC mnemonic in more than half of the reviews reinforced its usefulness in formulating the question, facilitating the identification of essential elements and the applicability of the findings^9^. It is noteworthy that the incorporation of the PCC contributes to the development of a clearer objective, more focused on the object of study^9^ ^,^ ^20^.

The application of filters and limiters in searches was common practice. However, few authors justified these choices. This methodological silence can introduce bias and limit reproducibility, especially when language or date restrictions are made without explicit reasons^5^ ^,^ ^21^. In the Brazilian context - marked by cultural and linguistic diversity - justifying language and time filters is essential to avoid excluding relevant regional evidence.

The three-step search strategy, as recommended by the JBI^9^, and the peer review of search strategies, as advocated by the PRESS guideline^22^, were rarely mentioned, which may indicate operational limitations or limitations in researcher training^5^ ^,^ ^8^ ^,^ ^22^. Complementary practices such as manual searching and reference scanning were also underutilized, which may have reduced the scope of studies^23^.

Most Brazilian scoping reviews analyzed between 21 and 50 studies, which indicated a sufficient approach for analytical robustness without sacrificing data depth^22^. Leaner or very extensive syntheses were less frequent, suggesting a preference for focused and manageable scopes^23^.

Regarding data extraction and analysis, the authors employed diverse strategies, with forms adapted from the JBI predominating. Only a small proportion reported the use of assistive technologies, such as qualitative analysis software or automated spreadsheets, revealing an opportunity to expand the adoption of digital resources^11^ ^,^ ^14^ ^,^ ^24^ ^-^ ^26^. The same pattern was observed in the presentation of results, mostly through tables, graphs, and thematic categories. The use of more interactive resources, such as dashboards or dynamic visualizations, was still incipient, limiting the communicative reach of the mappings.

In summary, although researchers have made progress in incorporating methodological guidelines, a gap persisted between scientific rigor and technological innovation. The predominance of manual processes, the low rate of protocol registrations, and the underutilization of specialized software highlighted challenges to be overcome to increase the consistency and quality of scoping reviews^25^. This study had limitations related to the limited databases searched, which may have reduced the scope of the results, and the exclusion of reviews conducted in other disciplinary or international contexts, limiting the generalizability of the findings. Despite these limitations, it offered relevant contributions to nursing practice and research in Brazil by promoting methodological standardization and encouraging the adoption of technologies that strengthen evidence-based practice.

By providing an updated overview of the development of scoping reviews conducted by Brazilian nurses, this study emphasized the importance of continuing education, with a focus on methodological qualifications and technology integration^10^. The systematic dissemination of updated guidelines (JBI and PRISMA-ScR), combined with innovation initiatives - such as the use of qualitative analysis software and AI for screening - has the potential to strengthen scientific production in nursing and expand the usefulness of these reviews in clinical, educational, and managerial practice. The experimental adoption of artificial intelligence in the categorization stage, carried out in this study, demonstrated feasibility and efficiency, constituting a promising path to qualify future scoping reviews conducted by Brazilian nurses. According to the methodological guidance recently published by the JBI group, which describes four progressive levels of automation and recommends recording the chosen tools in the protocol, with prior piloting and training, the incorporation of AI must be accompanied by formal validation and transparent reporting to ensure traceability and methodological safety^24^. Subsequent research should evaluate the impact of this technology on execution time, completeness, and clarity of results, as well as explore its applicability in different areas of nursing.

## Conclusion

This study showed that, in scoping reviews conducted by Brazilian nurses, data were mostly extracted using adapted JBI forms, with little use of supporting software; the analysis focused on descriptive summaries and thematic categorization; and the presentation of results favored charts and tables, rarely resorting to interactive visualizations.

This panorama reinforces the importance of standardized guidelines (JBI, PRISMA-ScR) and advanced technologies - including AI for screening and dashboards for dissemination - to increase the transparency, replicability, and clinical impact of scoping reviews. Investments in continuing education should focus not only on adherence to methodological guidelines, but also on training in digital tools that optimize the extraction, analysis, and communication of findings. Future research should evaluate (i) the effect of AI use on the speed and completeness of data extraction, (ii) the contribution of qualitative software to analytical depth, and (iii) the value of interactive visualizations for decision-making in Brazilian nursing practice and management scenarios.

## Data Availability

All data generated or analysed during this study are included in this published article.
