# Supplementary material for: Extraction, analysis and presentation of results in a scoping review in Brazilian nursing: scoping review
Source: Rev Lat Am Enfermagem. 2026 Mar 16;34:e4786. doi: 10.1590/1518-8345.7832.4786 (PMC12991387; doi:10.1590/1518-8345.7832.4786)
Supplement: Translation

# Translation: Extração, análise e apresentação de resultados em revisão de escopo na enfermagem brasileira: revisão de escopo

**DOI:** 10.1590/1518-8345.7832.4787

**Authors:** Isabelle Cristinne Pinto Costa, Patricia Treviso, Lucélia Terra Chini, Murilo César do Nascimento, Andreia Cristina Barbosa Costa, Patrícia Scotini Freitas, Karina Dal Sasso Mendes

**Affiliations:** 1 Universidade Federal de Alfenas, Escola de Enfermagem, Alfenas, MG, Brasil.; 2 Universidade Federal do Rio Grande do Sul, Escola de Enfermagem, Porto Alegre, RS, Brasil.; 3 Universidade de São Paulo, Escola de Enfermagem de Ribeirão Preto, Centro Colaborador da OPAS/OMS para o Desenvolvimento da Pesquisa em Enfermagem, Ribeirão Preto, SP, Brasil.

**Keywords:** Enfermagem, Análise de Dados, Brasil, Prática Clínica Baseada em Evidências, Enfermagem Baseada em Evidências, Revisão de Escopo.

## Abstract

**Objetivo:**

mapear a literatura sobre extração, análise e apresentação de resultados em revisões de escopo na enfermagem brasileira.

**Método:**

revisão de escopo conforme diretrizes do Instituto Joanna Briggs, nas fontes: PubMed, Literatura Latino-Americana e do Caribe em Ciências da Saúde, CINAHL, Embase, Scopus, *Web of Science Core Collection*, *Cochrane Database of Systematic Reviews* e *Google Scholar*. A busca foi estruturada em três etapas e executada por dois revisores independentes.

**Resultados:**

cento e quarenta e três estudos foram incluídos; o número anual subiu de 1 (2013) para 41 (2023). No título, 90,2% declararam “revisão de escopo”; 74,1% seguiram o referencial JBI, mas apenas 60,8% citaram o PRISMA-ScR e 39,2 % registraram protocolo. A extração manteve-se majoritariamente manual: 82,5% usaram formulários JBI adaptados e só 11,9% recorreram a *softwares* de apoio. Predominaram sínteses descritivas e categorização temática. A apresentação privilegiou quadros e tabelas (96%).

**Conclusão:**

as revisões de escopo em Enfermagem no Brasil utilizam procedimentos padronizados, mas carecem de maior adesão ao PRISMA-ScR, registro prévio e tecnologias digitais. Capacitação em guias internacionais, IA e visualizações interativas são prioritárias para ampliar transparência, reprodutibilidade e impacto clínico.

## Destaques

**(1)** Mapeamento de práticas de extração e análise em revisões de escopo na enfermagem. **(2)** Crescimento das revisões de escopo na última década, com 143 estudos incluídos. **(3)** Diretrizes metodológicas seguidas; tecnologias e IA ainda pouco exploradas. **(4)** Baixa adoção do PRISMA-ScR indica necessidade de maior capacitação.**(5)** Revisões de escopo fortalecem evidências para a prática na enfermagem brasileira.

## Introdução

A síntese de evidências abrange diversos tipos de revisões, sendo as revisões de escopo uma abordagem cada vez mais adotada para sumarizar evidências em diferentes campos. Esse tipo de revisão ganhou popularidade a partir de 2012^1^ ^-^ ^2^.

O primeiro guia metodológico para revisões de escopo foi publicado por Arksey e O’Malley em 2005, oferecendo uma estrutura inicial baseada em suas observações e reflexões sobre estudos de escopo existentes. Em 2010, Levac e colaboradores propuseram aprimoramentos nessa estrutura^3^. Posteriormente, em 2014, o Instituto Joanna Briggs (JBI) e sua rede de colaboradores conduziram uma extensa revisão da literatura, resultando em recomendações publicadas sobre a elaboração de revisões de escopo^4^ ^-^ ^6^. Essas diretrizes vêm sendo refinadas em sucessivos guias metodológicos de apoio a pesquisadores, elaborados por especialistas em revisões de escopo e publicados em 2017, 2020 e, mais recentemente, em 2024^7^ ^-^ ^9^.

Segundo o JBI, uma revisão de escopo é uma síntese de evidências voltada a mapear, de forma sistemática e abrangente, a extensão e os tipos de estudos disponíveis sobre um tema, conceito ou pergunta clínica, reunindo pesquisas primárias, revisões e outras fontes pertinentes^2^.

Para realizá-la com rigor, é indispensável elaborar um protocolo prévio que detalhe objetivo, questões de pesquisa e critérios de elegibilidade, bem como adotar uma estratégia de busca ampla seguida de triagem minuciosa. Recomenda-se a participação de, pelo menos, dois revisores independentes e a observância de diretrizes específicas - em especial o guia de relato *Preferred Reporting Items for Systematic Reviews and Meta-Analyses for Scoping Reviews* (PRISMA-ScR), que padroniza o reporte e reforça a transparência do processo^1^ ^,^ ^10^.

A etapa de extração e análise de dados em revisões de escopo passou a ser tratada com maior detalhe após a publicação, em 2023, de recomendações específicas do JBI que padronizam formulários, estratégias de codificação e formas de síntese^11^.

No Brasil, o distanciamento social imposto pela COVID-19 acelerou essa agenda: diante do volume de estudos emergentes, muitos grupos de pesquisa recorreram às revisões de escopo para organizar rapidamente as evidências e embasar decisões clínicas e de política pública. A flexibilidade do método - capaz de abarcar diferentes desenhos de estudo e fontes de informação - mostrou-se particularmente útil no contexto pandêmico.

À medida que a metodologia avança, torna-se imprescindível que enfermeiros adotem as diretrizes mais recentes, sobretudo quando os resultados se destinam a orientar novas investigações ou a subsidiar práticas assistenciais^12^.

Em março de 2023, foi conduzida uma busca exploratória no *Open Science Framework* (OSF), *PubMed* e *JBI Evidence Synthesis* para identificar revisões de escopo - publicadas ou em andamento - sobre extração, análise e apresentação de resultados em revisões de escopo na Enfermagem. Não se localizaram revisões com esse escopo. Foram recuperados apenas (i) um artigo metodológico que discute diretrizes para revisões de escopo em Enfermagem e Obstetrícia^12^ e (ii) uma revisão sistemática publicada em 2022 que descreveu características metodológicas de revisões de escopo em periódicos de Enfermagem, apontando inconsistências nas etapas de análise e apresentação dos dados e recomendando o uso do *framework* PAGER para padronizar o relato dos achados^13^ ^-^ ^14^. Esses achados evidenciam a lacuna que o presente estudo se propõe a preencher.

A ausência de sínteses que abordem, de forma específica, os procedimentos de extração, análise e apresentação de resultados em revisões de escopo conduzidas por pesquisadores de Enfermagem no Brasil evidencia uma lacuna metodológica. Diante disso, a presente revisão de escopo tem por objetivo mapear a literatura sobre extração, análise e apresentação de resultados em revisões de escopo no contexto da enfermagem brasileira.

## Método

### Protocolo e registro

Este estudo foi conduzido conforme a metodologia estabelecida pelo *Joanna Briggs Institute* (JBI) para revisões de escopo. As etapas seguidas incluíram: definição e alinhamento do objetivo e da pergunta de pesquisa; desenvolvimento dos critérios de inclusão alinhados com o objetivo; descrição da abordagem para a busca, seleção, extração e apresentação das evidências; e a execução dessas etapas com subsequente análise e resumo das evidências, destacando as conclusões e implicações das descobertas^9^. A metodologia foi escolhida por permitir um mapeamento abrangente das evidências, adequado para os objetivos deste estudo. O protocolo foi registrado no repositório OSF em 02 de maio de 2023^15^, e o guia PRISMA-ScR foi utilizado para relatar os resultados^1^.

### Critérios de elegibilidade

Esta revisão abordou a seguinte questão de pesquisa, estruturada pelo mnemônico População, Conceito e Contexto (PCC): como as revisões de escopo têm sido conduzidas em relação à extração, análise e apresentação de resultados no contexto da enfermagem brasileira? 

A população deste estudo incluiu publicações que utilizaram a revisão de escopo como método principal. Foram excluídos estudos que empregaram metodologias diferentes, mesmo que a revisão de escopo fosse uma etapa do processo de pesquisa. No que se refere ao conceito, considerou-se os estudos que descreveram a condução da extração, análise e apresentação de resultados em revisões de escopo. O foco foi na forma como esses processos foram descritos e executados pelos pesquisadores. Em relação ao contexto, a revisão abrangeu estudos realizados no âmbito da enfermagem brasileira, com pelo menos um enfermeiro como autor. Foram excluídos estudos de outras áreas da saúde ou que envolvessem países latino-americanos além do Brasil.

Estudos publicados em qualquer idioma foram incluídos. A revisão abrangeu publicações a partir de 2005 até fevereiro de 2024, delimitando o surgimento das primeiras diretrizes metodológicas^16^.

### Fontes de informação

Após a validação do protocolo, foram realizadas buscas em várias fontes de dados, selecionadas por sua relevância na área de saúde e enfermagem. As seguintes fontes foram utilizadas: Literatura Latino-Americana e do Caribe em Ciências da Saúde (LILACS), mantida pela Biblioteca Virtual em Saúde (BVS); *PubMed*, repositório de literatura biomédica da *The United States National Library of Medicine* (NLM); *The Cumulative Index to Nursing and Allied Health Literature* (CINAHL), fonte essencial para literatura em enfermagem, publicada pela EBSCO *Information Services*; Embase, agregada pela *Elsevier*; Scopus, um dos maiores bancos de dados multidisciplinares, também agregada pela *Elsevier*; *Cochrane Database of Systematic Reviews*, publicada pela *Cochrane Collaboration* e acessível via *Wiley Online Library*; *Web of Science Core Collection* (WOSCC), mantida pela *Clarivate Analytics*.

Para identificar literatura cinza, foram realizadas pesquisas no *Google Scholar*. 

### Busca na literatura

A estratégia de busca foi desenvolvida e implementada em três etapas distintas:

- Busca preliminar: envolveu a realização de uma busca preliminar nas bases de dados CINAHL e LILACS, com o objetivo de avaliar a viabilidade da revisão proposta, além de validar e alinhar os termos a serem utilizados na estratégia de busca. Foram identificados descritores controlados e sinônimos presentes nos Tesauros dos Descritores em Ciências da Saúde (DeCS/MeSH), dos Títulos CINAHL e também do *Emtree terms*. Além disso, foram analisadas as palavras-chave contidas nos títulos e resumos dos artigos relevantes analisados.

- Refinamento dos termos e construção da estratégia: com base nas informações coletadas na etapa preliminar, a segunda etapa consistiu na estratégia de busca final para a *PubMed*. A estratégia incluiu todas as palavras-chave e termos indexados relevantes contidos no *Medical Subject Headings* (MeSH), somados aos termos identificados na etapa anterior, sendo desenvolvida com a contribuição da equipe de estudo e revisada por um bibliotecário especializado para garantir sua precisão e abrangência.

- Adaptação para outras fontes de informação: na terceira etapa, a estratégia de busca elaborada para a *PubMed* foi adaptada para as outras fontes de informação selecionadas. Isso envolveu alguns ajustes dos comandos para adequação à sintaxe específica de cada fonte de informação e o uso de operadores booleanos AND e OR para combinar termos controlados e não controlados, de forma a otimizar a recuperação de estudos relevantes em cada fonte.

Decidiu-se não realizar a busca manual nas listas de referências dos estudos incluídos, tendo em vista que a amostra final foi considerada suficientemente ampla e representativa, o que permitiu uma análise completa e detalhada da produção científica sobre o tema.

A busca foi implementada em 20 de fevereiro de 2024. As estratégias de busca foram adaptadas às fontes de informação, conforme exemplo da busca realizada na *PubMed*, descritas a seguir: (“Scoping review”[Title/Abstract] OR “Literature Mapping”[Title/Abstract] OR “Mapping Review”[Title/Abstract] OR “Mapping of Research”[Title/Abstract] OR “Scoping Report”[Title/Abstract] OR “Scoping Study”[Title/Abstract] OR “Preliminary review”[Title/Abstract] OR “Scope review”[Title/Abstract] OR “Comprehensive review”[Title/Abstract] OR “Mapping study”[Title/Abstract] OR “Preliminary investigation”[Title/Abstract] OR “Evidence mapping”[Title/Abstract]) AND (“Nursing”[MeSH Terms] OR “Nursing”[Title/Abstract] OR “Nursings”[Title/Abstract] OR “Nurses”[MeSH Terms] OR “Nurses”[Title/Abstract] OR “Nurse”[Title/Abstract] OR “Registered Nurse”[Title/Abstract]) AND (“Brazil”[MeSH Terms] OR “Brazil”[Title/Abstract] OR “Brazil”[All Fields] OR “Brazilian”[All Fields] OR “Brasilian”[All Fields]).

### Seleção dos estudos

Após a realização das buscas nas fontes de informação, todos os registros identificados foram agrupados e carregados no *software EndNote* 20, onde as duplicatas foram removidas^17^. Em seguida, os registros foram exportados para o aplicativo *online Rayyan*, utilizado para a etapa de seleção dos estudos^18^. Dois revisores independentes, trabalhando de forma individual, analisaram os títulos e resumos para avaliar a conformidade com os critérios de seleção previamente estabelecidos.

Os textos completos dos estudos potencialmente relevantes foram avaliados em detalhes por dois revisores independentes, também de forma individual, de acordo com os critérios de inclusão. Os motivos de exclusão de quaisquer estudos cujo texto completo não atendeu aos critérios de inclusão foram registrados e relatados na revisão de escopo final. Quaisquer divergências entre os revisores, em qualquer etapa do processo de seleção dos estudos, foram resolvidas por meio de discussão ou pela intervenção de um terceiro revisor.

### Extração e análise de dados

Os dados foram extraídos dos estudos incluídos por dois revisores independentes, a fim de minimizar erros e vieses. Foi utilizado um roteiro de extração de dados proposto pelo JBI^14^, que foi adaptado pelos revisores e desenvolvido no *Microsoft Excel*.

Os dados extraídos incluíram informações detalhadas sobre a população, o conceito, o contexto^8^. O formulário de extração foi estruturado para capturar informações específicas, como a identificação dos autores, título, ano, idioma e periódico de publicação, além da instituição do primeiro autor e o país de origem do periódico. Também foram extraídos dados sobre o objetivo do estudo, a clareza na identificação da revisão de escopo como metodologia utilizada, o tema abordado e o referencial metodológico seguido. Adicionalmente, o formulário verificou se as etapas metodológicas foram descritas e se o estudo aderiu ao guia PRISMA-ScR, incluindo o uso do fluxograma e o registro do protocolo da revisão. Também observou-se se a estratégia de busca foi documentada detalhadamente, com ênfase no uso do mnemônico PCC, definição da pergunta de pesquisa, critérios de elegibilidade e aplicação de filtros ou limitadores^8^ ^,^ ^14^.

A extração dos dados abrangeu tanto informações bibliométricas quanto elementos do PCC, considerando o uso de *softwares* como *EndNote* e *Rayyan* para auxiliar na seleção dos estudos. Para a análise dos dados buscou-se identificar se foi descrita em termos de métodos descritivos e quantitativos, com o uso de gráficos e tabelas. O número de estudos incluídos, as limitações identificadas e as lacunas de conhecimento apontadas pelos autores também foram relatados. Um formulário de orientação de extração de dados foi desenvolvido para acompanhar o formulário principal, detalhando cada item a ser extraído e compartilhado com os revisores.

Na extração de dados, foi realizado um teste-piloto, no qual cada revisor completou a extração de dados de, pelo menos, três estudos de forma independente. Durante o teste-piloto, os revisores avaliaram o formulário respondendo a questões sobre a ausência de itens, redundância, clareza e tempo necessário para a extração de dados. Essas reflexões ajudaram a ajustar o tempo e a estrutura da extração^11^.

A equipe de revisão de escopo realizou reuniões regulares para facilitar a comunicação durante todo o processo de extração de dados, que foi um processo interativo para garantir que atendesse aos objetivos da revisão. 

Embora não tenha sido necessário, estava previsto o contato com os autores dos estudos incluídos para solicitar dados ausentes ou adicionais.

### Resumo ou sumário dos resultados

Na análise dos dados desta revisão de escopo, utilizou-se uma abordagem mista, combinando métodos qualitativos e quantitativos. Inicialmente, uma codificação aberta foi realizada, permitindo que os pesquisadores identificassem temas e conceitos relevantes diretamente dos dados brutos de forma imparcial. A partir dessa codificação, foram desenvolvidas categorias preliminares que refletiram as principais áreas de interesse do estudo. 

Devido ao quantitativo de estudos incluídos na amostra, foi necessário utilizar o suporte da inteligência artificial (IA), como o ChatGPT 4.0, para auxiliar no processo de categorização. Com o auxílio da IA, foi possível otimizar a identificação de padrões e tendências, facilitando o agrupamento e refinamento das categorias com maior precisão. O conteúdo gerado com o auxílio do ChatGPT 4.0 foi submetido à validação criteriosa por dois revisores independentes, com experiência na condução de revisões de escopo e familiaridade com métodos de análise qualitativa. 

O processo de categorização seguiu os princípios da análise de conteúdo qualitativa básica, conforme recomendado nas diretrizes atualizadas do JBI para revisões de escopo^11^, incluindo as etapas de codificação aberta, desenvolvimento de categorias e revisão cruzada. 

O *prompt* utilizado para auxiliar na categorização foi o seguinte: “Aja como um especialista em revisão de escopo. A seguir, será apresentada uma tabela com títulos e resumos de estudos incluídos. Com base no conteúdo, identifique temas emergentes e agrupe os estudos em categorias temáticas, justificando cada agrupamento com base na similaridade conceitual”.

Os dados não foram enviados via *upload* direto para a IA, mas sim organizados em uma tabela com as informações dos estudos (autores, título, resumo e palavras-chave), e inseridos manualmente no ambiente seguro da ferramenta ChatGPT 4.0. 

O conteúdo gerado foi tratado como apoio à codificação inicial, e não como categorização final. A validação final das categorias e subcategorias foi realizada pela equipe de pesquisadores, com registro das decisões em ata e planilhas de rastreabilidade. Esse processo visou tornar a etapa de análise mais eficiente e transparente, sem comprometer o rigor metodológico. As categorias derivadas foram posteriormente discutidas em reuniões da equipe, garantindo o princípio da triangulação dos dados. A utilização da IA foi, portanto, complementar, assistida e supervisionada, e não substitutiva à análise humana.

Paralelamente, foi conduzida uma análise quantitativa simples, na qual as frequências e porcentagens foram calculadas para descrever a distribuição dos dados em relação às variáveis-chave, como temas abordados e características demográficas dos estudos. Além disso, com o auxílio da IA, ficou mais fácil a elaboração de figuras, que foram geradas a partir do CANVA, tornando a visualização dos resultados mais clara e acessível. Essas representações visuais facilitaram a interpretação dos resultados, proporcionando uma visão abrangente e clara das evidências coletadas.

Os resultados do processo de seleção dos estudos foram sintetizados em um fluxograma PRISMA-ScR^1^. 

## Resultados

Um total de 602 registros foram identificados nas bases *PubMed* (n=132), LILACS (n=62), CINAHL (n=55), Embase (n=150), Scopus (n=57), WOSCC (n=41), *Cochrane* (n=5) e *Google Scholar* (n=100). Após a exportação para o *EndNote*, 160 registros duplicados foram removidos, resultando em 442 estudos que seguiram para o processo de triagem no *Rayyan*. Na primeira fase do rastreamento, realizada por título e resumo, 262 registros foram excluídos, o que levou 180 estudos para a fase de leitura na íntegra, sendo que dois não foram recuperados. Nessa etapa, ainda foram excluídos 35 estudos após a leitura completa. Assim, 143 estudos foram incluídos para extração e análise de dados, conforme foi ilustrado na Figura 1.

**Figura 1:** Fluxograma de identificação, seleção e inclusão de estudos da revisão de escopo conforme diretrizes do PRISMA-ScR^1^. Alfenas, MG, Brasil, 2024

A distribuição dos estudos por ano de publicação revelou tendências significativas sobre a evolução da produção científica relacionada às revisões de escopo. O maior pico de publicações ocorreu em 2023 (41; 28,7%), seguido por 2021 (36; 25,2%) e 2022 (30; 21,0%). Houve também uma produção relevante em 2020 (14; 9,8%) e 2019 (9; 6,3%). Os anos anteriores apresentaram uma menor quantidade de publicações, como em 2018 (4; 2,8%), 2017 (2; 1,4%), 2016 (1; 0,7%) e 2015 (3; 2,1%). Até a data da busca nas fontes de informação, três (2,1%) estudos foram identificados para o ano de 2024.

A análise dos idiomas de publicação dos 143 estudos revelou uma significativa diversidade linguística na produção científica. O idioma inglês se destacou como o mais utilizado, com 38 (26,6%) estudos publicados exclusivamente neste idioma. A combinação de “inglês, português e espanhol” apareceu como a mais frequente, com 46 (32,2%) publicações. O uso combinado de “português e inglês” também foi expressivo, com 39 (27,3%) estudos. O português foi o idioma exclusivo de 18 (12,6%) estudos, enquanto o espanhol foi exclusivo de apenas um (0,7%) estudo, assim como a combinação de “português e espanhol”, que também apareceu em uma (0,7%) publicação.

A Figura 2 ilustrou os procedimentos metodológicos adotados em revisões de escopo realizadas por enfermeiros no Brasil, destacando o uso de várias práticas e tendências importantes. A maioria dos estudos (140; 97,9%) identificou claramente em seus métodos que se tratava de uma revisão de escopo, sendo que 129 (90,2%) fizeram essa identificação diretamente no título do estudo. O verbo “mapear” foi o mais utilizado, aparecendo em 53 objetivos, o que representou 37,1% do total. O referencial teórico mais empregado foi o JBI, utilizado em 106 (74,1%) estudos, seguido por Arksey e O’Malley, com 10 (7,0%) estudos. Além disso, uma combinação do JBI com as orientações de Arksey e O’Malley foi utilizada em cinco (3,5%) estudos. A maioria dos estudos (87; 60,8%) fez uso do PRISMA-ScR para descrever suas pesquisas. Uma parcela significativa dos estudos (92; 64,3%) adotou uma abordagem estruturada, com a definição clara das etapas específicas da revisão de escopo. No entanto, apenas 56 (39,2%) dos estudos mencionaram o registro do protocolo. Entre os estudos, 116 (81,1%) utilizaram o mnemônico PCC para estruturar suas revisões de escopo. A questão de pesquisa foi mencionada em 137 (95,8%) estudos, enquanto apenas 30 (21,0%) incluíram subquestões de pesquisa.

**Figura 2:** Panorama metodológico das revisões de escopo na enfermagem brasileira. Alfenas, MG, Brasil, 2024

A grande maioria dos estudos (106; 74,1%) mencionou o uso de filtros e limitadores em suas estratégias de busca. O filtro mais utilizado foi a combinação de recorte temporal e idioma, aplicada em 32 (30,2%) estudos. Em seguida, o filtro por idioma isolado foi mencionado em 28 (26,4%) estudos. Outros filtros foram relatados, incluindo: recorte temporal isolado em 9 (8,5%) estudos, combinação de filtros por idioma e tipos de fontes em 5 (4,7%) estudos, e filtro por documentos disponíveis na íntegra combinado com idioma, presente em 6 (5,7%) estudos. Filtros menos frequentes abrangeram a combinação de idioma, fontes e documentos disponibilizados na íntegra em 1 (0,9%) estudo, além de combinações envolvendo recorte temporal e fontes, ou recorte temporal e documentos na íntegra, cada uma mencionada em 1 (0,9%) estudo. O filtro que envolveu apenas tipos de fontes foi utilizado em 3 (2,8%) estudos. Além disso, 6 (5,7%) estudos não detalharam as especificidades dos filtros utilizados.

Apenas 14 (9,8%) estudos relataram a realização de um teste-piloto da estratégia de busca. A estratégia de busca foi desenvolvida em três etapas em 44 (30,8%) estudos. Mais da metade dos estudos (85; 59,4%) utilizou a literatura cinzenta como fonte de informação, e a busca manual em fontes específicas foi mencionada em 26 (18,2%) estudos. A busca pelas referências dos estudos incluídos foi empregada em 79 (55,2%) estudos. A estratégia de busca foi apresentada na maioria dos estudos (105; 73,4%). A seleção dos estudos por título e resumo (Fase 1) e por leitura na íntegra (Fase 2) foi amplamente praticada, com 115 (80,4%) e 116 (81,1%) dos estudos indicando essas fases, respectivamente. A seleção por dois revisores foi mencionada em 105 (73,4%) dos estudos.

Quanto à distribuição dos estudos selecionados e incluídos nas revisões de escopo, categorizando-os em quatro intervalos distintos com base no número de estudos, os intervalos baixos e moderados (1-50 estudos) dominaram a distribuição das revisões de escopo, representando, juntos, 117 (81,9%) estudos. Revisões que envolveram um número maior de estudos (51-100) foram menos comuns, com 18 (12,5%) estudos, enquanto revisões extremas (mais de 100 estudos) foram bastante raras, somando apenas 5,6%.

A Figura 3 abordou o processo de extração de dados. Primeiramente, observou-se que 57 (39,9%) estudos adotaram o formulário do JBI, um dos padrões mais reconhecidos para a extração sistemática de dados. Em relação à inclusão de elementos do PCC nos formulários de extração, 73 (51%) dos estudos mencionaram explicitamente sua aplicação. Outro ponto relevante foi a adesão aos protocolos planejados. Apenas um (0,7%) reportou desvio de protocolo na extração de dados. No que tange ao uso de tecnologias, apenas 17 (11,9%) estudos mencionaram explicitamente o uso de softwares para a extração de dados, como *Microsoft Excel,* ATLAS.ti^®^, *web*QDA e *Apple Numbers*. Quanto ao número de pesquisadores envolvidos na extração de dados, 70 (49%) estudos descreveram essa informação.

**Figura 3:** Itens dos formulários e *softwares* utilizados no processo de extração dos dados das revisões de escopo na enfermagem brasileira. Alfenas, MG, Brasil, 2024

A Figura 4 ofereceu uma visão abrangente sobre o processo de análise de dados, destacando tanto os softwares utilizados quanto os métodos de análise empregados. Observou-se que os principais softwares empregados para a análise de dados incluíram ferramentas qualitativas como ATLAS.ti^®^, MAXQDA, *web*QDA, e IRaMuTeQ, além de *softwares* de uso mais geral, como *Microsoft Excel* e *Apple Numbers*. Esses *softwares* desempenharam um papel fundamental na organização, codificação e análise sistemática dos dados. Quanto às abordagens metodológicas adotadas, verificou-se que a análise descritiva simples, bem como a análise temática e de conteúdo foram amplamente utilizadas para organizar e categorizar os dados, respectivamente.

**Figura 4:** Processo e *softwares* de análise dos dados das revisões de escopo na enfermagem brasileira. Alfenas, MG, Brasil, 2024

A Figura 5 destacou as principais formas de apresentação dos resultados em revisões de escopo no Brasil, com uma predominância do uso de quadros e categorias temáticas, que organizaram e sintetizaram informações complexas, facilitando comparações e a identificação de padrões. Tabelas e gráficos também foram amplamente utilizados.

**Figura 5:** Formas de apresentação dos resultados em revisões de escopo na enfermagem brasileira. Alfenas, MG, Brasil, 2024

As revisões de escopo examinadas evidenciaram uma expressiva diversidade de temas, agrupáveis em três eixos principais: clínico-assistencial, populacional e educacional-político. No campo clínico, destacaram-se produções voltadas a cuidados paliativos, saúde mental, segurança do paciente, infecções associadas à assistência e processos diagnósticos de enfermagem.

Em relação a abordagens emergentes, houve ênfase crescente em práticas avançadas de enfermagem, tecnologias digitais aplicadas à formação e assistência, bem como enfermagem forense. No plano populacional, os estudos contemplaram grupos em situação de vulnerabilidade, como idosos, pessoas LGBTI+, indígenas, privados de liberdade, migrantes e refugiados. Também se identificou investigações centradas em condições clínicas específicas - a exemplo de distúrbios hipertensivos na gestação, câncer, doença de Parkinson e infecções por COVID-19 em múltiplos perfis - além de tópicos como adesão terapêutica na tuberculose e estratégias de manejo da dor.

No domínio formativo e ético-político, foram abordadas metodologias ativas, simulação clínica, uso de jogos digitais, estilos de aprendizagem, direitos humanos no cuidado a pessoas com transtornos mentais e ações de preservação forense em contextos de violência. Ademais, surgiram análises relativas à gestão e formulação de políticas públicas, como modelos de organização em saúde mental, fiscalização profissional, sustentabilidade hospitalar e acesso a serviços em regiões de fronteira. Tal panorama temático reafirma a relevância das revisões de escopo na consolidação do conhecimento em enfermagem e na orientação de prioridades para futuras agendas de pesquisa.

## Discussão

Este é o primeiro mapeamento a quantificar e interpretar a evolução metodológica das revisões de escopo produzidas por enfermeiros brasileiros. A análise temporal revelou um crescimento expressivo das revisões de escopo publicadas por enfermeiros brasileiros nos últimos três anos (2021-2023). Tal expansão parece ter sido impulsionada por respostas a crises globais, incluindo a pandemia de COVID-19, bem como pela necessidade de abordagens abrangentes para questões de saúde complexas, características inerentes às revisões de escopo^19^. Como já sugerido pela literatura, a flexibilidade dessa abordagem em cenários de conhecimento em rápida mudança^15^ vem consolidando a metodologia no campo da Enfermagem nacional.

Entretanto, o avanço quantitativo não foi acompanhado por melhora proporcional na qualidade metodológica. Embora 90,2% dos estudos tenham indicado no título tratar-se de revisão de escopo e 74,1% tenham adotado o referencial JBI, apenas 60,8% utilizaram o guia PRISMA-ScR, e 39,2% mencionaram o registro de protocolo. Em quase dois quintos das publicações, portanto, persiste um risco relevante de baixa transparência e reprodutibilidade, cenário igualmente descrito em periódicos internacionais de Enfermagem^13^.

Esses achados revelam oportunidades de aprimoramento na formação de pesquisadores, sobretudo quanto à importância da adesão rigorosa às diretrizes metodológicas, com vistas a ampliar a robustez e a transparência das revisões e a maximizar seu impacto na prática clínica, educacional e gerencial^1^ ^,^ ^10^ ^,^ ^12^.

Embora a maioria dos estudos tenha descrito as etapas da revisão de escopo, uma parcela significativa omitiu detalhes relevantes do processo, o que evidenciou a necessidade de maior clareza e sistematização^8^ ^,^ ^13^. O emprego do mnemônico PCC em mais da metade das revisões reforçou sua utilidade na formulação da pergunta, facilitando a identificação de elementos essenciais e a aplicabilidade dos achados^9^. Vale ressaltar que a incorporação do PCC contribui para a elaboração de um objetivo mais claro e direcionado ao objeto de estudo^9^ ^,^ ^20^.

A aplicação de filtros e limitadores nas buscas foi prática comum. Contudo, poucos autores justificaram essas escolhas. Esse silêncio metodológico pode introduzir viés e limitar a reprodutibilidade, especialmente quando se restringem idiomas ou datas sem explicitar motivo^5^ ^,^ ^21^. Para o contexto brasileiro - marcado por diversidade cultural e linguística -, justificar filtros de idioma e tempo é fundamental para evitar a exclusão de evidências regionais relevantes.

A estratégia de busca em três etapas, conforme recomendada pela JBI^9^ e a revisão por pares das estratégias de busca, preconizada pela diretriz PRESS^22^ foram pouco referidas, o que pode sinalizar limitações operacionais ou de formação dos pesquisadores^5^ ^,^ ^8^ ^,^ ^22^. Práticas complementares como busca manual e varredura de referências também foram subutilizadas, o que pode ter reduzido a abrangência dos estudos^23^.

A maioria das revisões de escopo brasileiras analisou entre 21 e 50 estudos, o que sinalizou uma abordagem suficiente para robustez analítica sem sacrificar a profundidade dos dados^22^. Sínteses mais enxutas ou muito extensas foram menos frequentes, sugerindo preferência por escopos focados e gerenciáveis^23^.

Quanto à extração e à análise de dados, os autores empregaram estratégias diversas, predominando formulários adaptados do JBI. Apenas uma pequena parcela relatou o uso de tecnologias de apoio, como *softwares* de análise qualitativa ou planilhas automatizadas, revelando oportunidade de ampliar a adoção de recursos digitais^11^ ^,^ ^14^ ^,^ ^24^ ^-^ ^26^. O mesmo padrão foi observado na apresentação dos resultados, majoritariamente realizada por meio de quadros, gráficos e categorias temáticas. O emprego de recursos mais interativos, como *dashboards* ou visualizações dinâmicas ainda foi incipiente, limitando o alcance comunicativo dos mapeamentos.

Em síntese, embora os pesquisadores tenham avançado na incorporação de diretrizes metodológicas, persistiu uma lacuna entre rigor científico e inovação tecnológica. A predominância de processos manuais, a baixa taxa de registros de protocolo e a subutilização de *softwares* especializados evidenciaram desafios a serem superados para elevar a consistência e a qualidade das revisões de escopo^25^.

Este estudo apresentou limitações relacionadas à restrição das bases de dados pesquisadas, o que pode ter reduzido a abrangência dos resultados, e à exclusão de revisões conduzidas em outros contextos disciplinares ou internacionais, restringindo a generalização dos achados. Apesar desses limites, ofereceu contribuições relevantes para a prática e a pesquisa em Enfermagem no Brasil, ao promover a padronização metodológica e ao incentivar a adoção de tecnologias que fortaleçam a prática baseada em evidências.

Ao traçar um panorama atualizado do desenvolvimento das revisões de escopo conduzidas por enfermeiros brasileiros, este estudo enfatizou a importância da formação continuada, com foco em qualificação metodológica e integração de tecnologias^10^. A disseminação sistemática de guias atualizados (JBI e PRISMA-ScR), aliada a iniciativas de inovação - como o uso de *softwares* de análise qualitativa e IA para triagem - possui potencial para fortalecer a produção científica em Enfermagem e ampliar a utilidade dessas revisões na prática clínica, educacional e gerencial.

A adoção experimental de inteligência artificial na etapa de categorização, realizada neste trabalho, demonstrou viabilidade e eficiência, constituindo um caminho promissor para qualificar futuras revisões de escopo conduzidas por enfermeiros brasileiros. Conforme a orientação metodológica recém-publicada pelo grupo JBI, que descreve quatro níveis progressivos de automação e recomenda registrar no protocolo as ferramentas escolhidas, com piloto e treinamento prévios, a incorporação de IA deve vir acompanhada de validação formal e relato transparente para garantir rastreabilidade e segurança metodológica^24^. Investigações subsequentes deveriam avaliar o impacto dessa tecnologia sobre tempo de execução, exaustividade e clareza dos resultados, bem como explorar sua aplicabilidade em diferentes áreas da Enfermagem.

## Conclusão

Este estudo evidenciou que, nas revisões de escopo conduzidas por enfermeiros brasileiros, os dados foram majoritariamente extraídos por meio de formulários adaptados do JBI, com baixo uso de *softwares* de apoio; a análise concentrou-se em sínteses descritivas e categorização temática; e a apresentação dos resultados privilegiou quadros e tabelas, raramente recorrendo a visualizações interativas.

Esse panorama reforça a importância de diretrizes padronizadas (JBI, PRISMA-ScR) e de tecnologias avançadas - incluindo IA para triagem e *dashboards* para disseminação - a fim de elevar transparência, replicabilidade e impacto clínico das revisões de escopo. Investimentos em formação continuada deverão focar não só na adesão a guias metodológicos, mas também na capacitação para ferramentas digitais que otimizem extração, análise e comunicação dos achados.

Futuras investigações deverão avaliar (i) o efeito do uso de IA na rapidez e exaustividade da extração de dados, (ii) a contribuição de *softwares* qualitativos à profundidade analítica e (iii) o valor de visualizações interativas para a tomada de decisão em cenários de prática e gestão em Enfermagem brasileira.

## Declaração de Disponibilidade de Dados:

Todos os dados gerados ou analisados durante este estudo estão incluídos neste artigo publicado.

# Translation: Extracción, análisis y presentación de resultados en revisión de alcance en la enfermería brasileña: revisión de alcance

**DOI:** 10.1590/1518-8345.7832.4785

**Authors:** Isabelle Cristinne Pinto Costa, Patricia Treviso, Lucélia Terra Chini, Murilo César do Nascimento, Andreia Cristina Barbosa Costa, Patrícia Scotini Freitas, Karina Dal Sasso Mendes

**Affiliations:** 1 Universidade Federal de Alfenas, Escola de Enfermagem, Alfenas, MG, Brasil.; 2 Universidade Federal do Rio Grande do Sul, Escola de Enfermagem, Porto Alegre, RS, Brasil.; 3 Universidade de São Paulo, Escola de Enfermagem de Ribeirão Preto, Centro Colaborador de la OPS/OMS para el Desarrollo de la Investigación en Enfermería, Ribeirão Preto, SP, Brasil.

**Keywords:** Enfermería, Análisis de Datos, Brasil, Práctica Clínica Basada en la Evidencia, Enfermería Basada en la Evidencia, Revisión de Alcance.

## Abstract

**Objetivo:**

mapear la literatura sobre extracción, análisis y presentación de resultados en revisiones de alcance en la enfermería brasileña.

**Método:**

revisión de alcance conforme a las directrices del Instituto Joanna Briggs, incluyendo publicaciones, en las fuentes: *PubMed*, Literatura Latinoamericana y del Caribe en Ciencias de la Salud, CINAHL, Embase, Scopus, *Web of Science Core Collection*, *Cochrane Database of Systematic Reviews* y *Google Scholar*. La búsqueda se estructuró en tres etapas y fue ejecutada por dos revisores independientes.

**Resultados:**

ciento cuarenta y tres estudios fueron incluidos; el número anual subió de 1 (2013) a 41 (2023). En el título, 90,2% declaró “revisión de alcance”; 74,1% siguió el marco JBI, pero solo 60,8% citó PRISMA-ScR y 39,2% registró protocolo. La extracción se mantuvo mayoritariamente manual: 82,5% usó formularios JBI adaptados y solo 11,9% recurrió a *software* de apoyo. Predominaron síntesis descriptivas y categorización temática. La presentación privilegió cuadros y tablas (96%).

**Conclusión:**

las revisiones de alcance en enfermería en Brasil utilizan procedimientos estandarizados, pero carecen de mayor adhesión a PRISMA-ScR, registro previo y tecnologías digitales. La capacitación en guías internacionales, IA y visualizaciones interactivas es prioritaria para ampliar transparencia, reproducibilidad e impacto clínico.

## Destacados

**(1)** Mapeo de prácticas de extracción y análisis en revisiones de alcance en enfermería. **(2)** Crecimiento de las revisiones de alcance en la última década, con 143 estudios incluidos. **(3)** Directrices metodológicas seguidas; tecnologías e IA aún poco exploradas. **(4)** Baja adopción de PRISMA-ScR indica necesidad de mayor capacitación. **(5)** Las revisiones de alcance fortalecen las evidencias para la práctica en la enfermería brasileña.

## Introducción

La síntesis de evidencias abarca diversos tipos de revisiones, y las revisiones de alcance constituyen un enfoque cada vez más adoptado para sintetizar evidencias en distintos campos. Este tipo de revisión ganó popularidad a partir de 2012^1^ ^-^ ^2^.

La primera guía metodológica para revisiones de alcance fue publicada por Arksey y O’Malley en 2005, y ofreció una estructura inicial basada en sus observaciones y reflexiones sobre estudios de alcance existentes. En 2010, Levac y colaboradores propusieron mejoras en esa estructura^3^. Posteriormente, en 2014, el Instituto Joanna Briggs (JBI) y su red de colaboradores realizaron una extensa revisión de la literatura, que dio lugar a recomendaciones publicadas sobre la elaboración de revisiones de alcance^4^ ^-^ ^6^. Estas directrices se han ido perfeccionando en sucesivas guías metodológicas de apoyo a investigadores, elaboradas por especialistas en revisiones de alcance y publicadas en 2017, 2020 y, más recientemente, en 2024^7^ ^-^ ^9^.

Según el JBI, una revisión de alcance es una síntesis de evidencias orientada a mapear, de forma sistemática y exhaustiva, la amplitud y los tipos de estudios disponibles sobre un tema, concepto o pregunta clínica, reuniendo investigaciones primarias, revisiones y otras fuentes pertinentes^2^.

Para llevarla a cabo con rigor, es indispensable elaborar un protocolo previo que detalle objetivo, preguntas de investigación y criterios de elegibilidad, así como adoptar una estrategia de búsqueda amplia seguida de un cribado minucioso. Se recomienda la participación de, al menos, dos revisores independientes y la observancia de directrices específicas, en especial la guía de reporte *Preferred Reporting Items for Systematic Reviews and Meta-Analyses for Scoping Reviews* (PRISMA-ScR), que estandariza el reporte y refuerza la transparencia del proceso^1^ ^,^ ^10^.

La etapa de extracción y análisis de datos en revisiones de alcance pasó a tratarse con mayor detalle tras la publicación, en 2023, de recomendaciones específicas del JBI que estandarizan formularios, estrategias de codificación y formas de síntesis^11^.

En Brasil, el distanciamiento social impuesto por la COVID-19 aceleró esta agenda: ante el volumen de estudios emergentes, muchos grupos de investigación recurrieron a las revisiones de alcance para organizar rápidamente las evidencias y fundamentar decisiones clínicas y de política pública. La flexibilidad del método, capaz de abarcar distintos diseños de estudio y fuentes de información, se mostró particularmente útil en el contexto pandémico.

A medida que la metodología avanza, se vuelve imprescindible que los profesionales de enfermería adopten las directrices más recientes, sobre todo cuando los resultados se destinan a orientar nuevas investigaciones o a subsidiar prácticas asistenciales^12^.

En marzo de 2023 se realizó una búsqueda exploratoria en *Open Science Framework* (OSF), *PubMed* y JBI *Evidence Synthesis* para identificar revisiones de alcance, publicadas o en curso, sobre extracción, análisis y presentación de resultados en revisiones de alcance en Enfermería. No se localizaron revisiones con ese alcance. Se recuperaron únicamente (i) un artículo metodológico que discute directrices para revisiones de alcance en Enfermería y Obstetricia^12^ y (ii) una revisión sistemática publicada en 2022 que describió características metodológicas de revisiones de alcance en revistas de Enfermería, señalando inconsistencias en las etapas de análisis y presentación de los datos y recomendando el uso del *framework* PAGER para estandarizar el reporte de los hallazgos^13^ ^-^ ^14^. Estos hallazgos evidencian la brecha que el presente estudio se propone cubrir.

La ausencia de síntesis que aborden, de forma específica, los procedimientos de extracción, análisis y presentación de resultados en revisiones de alcance realizadas por investigadores de Enfermería en Brasil evidencia una laguna metodológica. Por ello, la presente revisión de alcance tiene por objetivo mapear la literatura sobre extracción, análisis y presentación de resultados en revisiones de alcance en el contexto de la enfermería brasileña.

## Método

### Protocolo y registro

Este estudio se condujo conforme a la metodología establecida por el *Joanna Briggs Institute* (JBI) para revisiones de alcance. Las etapas seguidas incluyeron: definición y alineación del objetivo y de la pregunta de investigación; desarrollo de los criterios de inclusión alineados con el objetivo; descripción del abordaje para la búsqueda, selección, extracción y presentación de las evidencias; y la ejecución de esas etapas con el subsiguiente análisis y síntesis de las evidencias, destacando las conclusiones e implicaciones de los hallazgos^9^. La metodología fue elegida por permitir un mapeo exhaustivo de las evidencias, adecuado a los objetivos de este estudio. El protocolo fue registrado en el repositorio OSF el 2 de mayo de 2023^15^, y la guía PRISMA-ScR se utilizó para reportar los resultados^1^.

### Criterios de elegibilidad

Esta revisión abordó la siguiente pregunta de investigación, estructurada por el mnemónico Población, Concepto y Contexto (PCC): ¿cómo se han conducido las revisiones de alcance en relación con la extracción, análisis y presentación de resultados en el contexto de la enfermería brasileña?

La población de este estudio incluyó publicaciones que utilizaron la revisión de alcance como método principal. Se excluyeron estudios que emplearon metodologías diferentes, incluso si la revisión de alcance constituía una etapa del proceso de investigación. En lo que se refiere al concepto, se consideraron los estudios que describieron la conducción de la extracción, análisis y presentación de resultados en revisiones de alcance. El foco estuvo en la forma en que esos procesos fueron descritos y ejecutados por los investigadores. En relación con el contexto, la revisión abarcó estudios realizados en el ámbito de la enfermería brasileña, con al menos un enfermero como autor. Se excluyeron estudios de otras áreas de la salud o que involucraran países latinoamericanos además de Brasil.

Se incluyeron estudios publicados en cualquier idioma. La revisión abarcó publicaciones desde 2005 hasta febrero de 2024, delimitando el surgimiento de las primeras directrices metodológicas^16^.

### Fuentes de información

Tras la validación del protocolo, se realizaron búsquedas en varias fuentes de datos, seleccionadas por su relevancia en el área de salud y enfermería. Se utilizaron las siguientes fuentes: Literatura Latinoamericana y del Caribe en Ciencias de la Salud (LILACS), mantenida por la Biblioteca Virtual en Salud (BVS); *PubMed*, repositorio de literatura biomédica de la *The United States National Library of Medicine* (NLM); *The Cumulative Index to Nursing and Allied Health Literature* (CINAHL), fuente esencial para literatura en enfermería, publicada por EBSCO *Information Services*; Embase, indexada por *Elsevier*; Scopus, una de las mayores bases de datos multidisciplinarias, también indexada por *Elsevier*; *Cochrane Database of Systematic Reviews*, publicada por la *Cochrane Collaboration* y accesible vía *Wiley Online Library*; *Web of Science Core Collection* (WOSCC), mantenida por *Clarivate Analytics*.

Para identificar literatura gris, se realizaron búsquedas en *Google Scholar*. 

### Búsqueda en la literatura

La estrategia de búsqueda se desarrolló e implementó en tres etapas distintas:

- Búsqueda preliminar: implicó la realización de una búsqueda preliminar en las bases de datos CINAHL y LILACS, con el objetivo de evaluar la viabilidad de la revisión propuesta, además de validar y alinear los términos que se utilizarían en la estrategia de búsqueda. Se identificaron descriptores controlados y sinónimos presentes en los tesauros de los Descriptores en Ciencias de la Salud (DeCS/MeSH), de los Títulos CINAHL y también de *Emtree terms*. Además, se analizaron las palabras clave contenidas en los títulos y resúmenes de los artículos relevantes analizados.

- Refinamiento de los términos y construcción de la estrategia: con base en la información recopilada en la etapa preliminar, la segunda etapa consistió en la estrategia de búsqueda final para *PubMed*. La estrategia incluyó todas las palabras clave y términos indexados relevantes contenidos en *Medical Subject Headings* (MeSH), sumados a los términos identificados en la etapa anterior, y fue desarrollada con la contribución del equipo de estudio y revisada por un bibliotecario especializado para garantizar su precisión y exhaustividad.

- Adaptación a otras fuentes de información: en la tercera etapa, la estrategia de búsqueda elaborada para *PubMed* se adaptó a las otras fuentes de información seleccionadas. Esto implicó algunos ajustes de los comandos para adecuación a la sintaxis específica de cada fuente de información y el uso de los operadores booleanos AND y OR para combinar términos controlados y no controlados, a fin de optimizar la recuperación de estudios relevantes en cada fuente.

Se decidió no realizar la búsqueda manual en las listas de referencias de los estudios incluidos, considerando que la muestra final se consideró suficientemente amplia y representativa, lo que permitió un análisis completo y detallado de la producción científica sobre el tema.

La búsqueda se implementó el 20 de febrero de 2024. Las estrategias de búsqueda se adaptaron a las fuentes de información, conforme el ejemplo de la búsqueda realizada en PubMed, descrito a continuación: (“Scoping review”[Title/Abstract] OR “Literature Mapping”[Title/Abstract] OR “Mapping Review”[Title/Abstract] OR “Mapping of Research”[Title/Abstract] OR “Scoping Report”[Title/Abstract] OR “Scoping Study”[Title/Abstract] OR “Preliminary review”[Title/Abstract] OR “Scope review”[Title/Abstract] OR “Comprehensive review”[Title/Abstract] OR “Mapping study”[Title/Abstract] OR “Preliminary investigation”[Title/Abstract] OR “Evidence mapping”[Title/Abstract]) AND (“Nursing”[MeSH Terms] OR “Nursing”[Title/Abstract] OR “Nursings”[Title/Abstract] OR “Nurses”[MeSH Terms] OR “Nurses”[Title/Abstract] OR “Nurse”[Title/Abstract] OR “Registered Nurse”[Title/Abstract]) AND (“Brazil”[MeSH Terms] OR “Brazil”[Title/Abstract] OR “Brazil”[All Fields] OR “Brazilian”[All Fields] OR “Brasilian”[All Fields]).

### Selección de los estudios

Tras la realización de las búsquedas en las fuentes de información, todos los registros identificados fueron agrupados y cargados en el *software EndNote* 20, donde se eliminaron los duplicados^17^. A continuación, los registros se exportaron a la aplicación en línea *Rayyan*, utilizada para la etapa de selección de los estudios^18^. Dos revisores independientes, trabajando de forma individual, analizaron los títulos y resúmenes para evaluar la conformidad con los criterios de selección previamente establecidos.

Los textos completos de los estudios potencialmente relevantes fueron evaluados en detalle por dos revisores independientes, también de forma individual, de acuerdo con los criterios de inclusión. Los motivos de exclusión de cualquier estudio cuyo texto completo no cumpliera los criterios de inclusión se registraron y se informaron en la revisión de alcance final. Cualquier discrepancia entre los revisores, en cualquier etapa del proceso de selección de los estudios, se resolvió mediante discusión o mediante la intervención de un tercer revisor.

### Extracción y análisis de datos

Los datos fueron extraídos de los estudios incluidos por dos revisores independientes, a fin de minimizar errores y sesgos. Se utilizó un formulario de extracción de datos propuesto por el JBI^14^, que fue adaptado por los revisores y desarrollado en *Microsoft Excel*.

Los datos extraídos incluyeron información detallada sobre la población, el concepto y el contexto^8^. El formulario de extracción se estructuró para capturar información específica, como la identificación de los autores, título, año, idioma y revista de publicación, además de la institución del primer autor y el país de origen de la revista. También se extrajeron datos sobre el objetivo del estudio, la claridad en la identificación de la revisión de alcance como metodología utilizada, el tema abordado y el marco metodológico seguido. Adicionalmente, el formulario verificó si las etapas metodológicas fueron descritas y si el estudio adhirió a la guía PRISMA-ScR, incluyendo el uso del diagrama de flujo y el registro del protocolo de la revisión. Asimismo, se observó si la estrategia de búsqueda fue documentada detalladamente, con énfasis en el uso del mnemónico PCC, la definición de la pregunta de investigación, los criterios de elegibilidad y la aplicación de filtros o limitadores^8^ ^,^ ^14^.

La extracción de los datos abarcó tanto información bibliométrica como elementos del PCC, considerando el uso de *software* como *EndNote* y *Rayyan* para auxiliar en la selección de los estudios. Para el análisis de los datos se buscó identificar si fue descrito en términos de métodos descriptivos y cuantitativos, con el uso de gráficos y tablas. El número de estudios incluidos, las limitaciones identificadas y las lagunas de conocimiento señaladas por los autores también fueron reportados. Se elaboró un formulario guía de extracción de datos para acompañar el formulario principal, detallando cada ítem a extraer y compartido con los revisores.

En la extracción de datos se realizó una prueba piloto, en la que cada revisor completó la extracción de datos de, al menos, tres estudios de forma independiente. Durante la prueba piloto, los revisores evaluaron el formulario respondiendo a cuestiones sobre ausencia de ítems, redundancia, claridad y tiempo necesario para la extracción de datos. Estas reflexiones ayudaron a ajustar el tiempo y la estructura de la extracción^11^.

El equipo de revisión de alcance realizó reuniones regulares para facilitar la comunicación durante todo el proceso de extracción de datos, que fue un proceso iterativo para garantizar que respondiera a los objetivos de la revisión.

Aunque no fue necesario, se preveía el contacto con los autores de los estudios incluidos para solicitar datos ausentes o adicionales.

### Resumen o sumario de los resultados

En el análisis de los datos de esta revisión de alcance se utilizó un enfoque mixto, combinando métodos cualitativos y cuantitativos. Inicialmente, se realizó una codificación abierta, lo que permitió a los investigadores identificar temas y conceptos relevantes directamente a partir de los datos brutos de forma imparcial. A partir de esa codificación, se desarrollaron categorías preliminares que reflejaron las principales áreas de interés del estudio.

Debido a la cantidad de estudios incluidos en la muestra, fue necesario utilizar el apoyo de la inteligencia artificial (IA), como ChatGPT 4.0, para auxiliar en el proceso de categorización. Con la ayuda de la IA, fue posible optimizar la identificación de patrones y tendencias, facilitando el agrupamiento y el refinamiento de las categorías con mayor precisión. El contenido generado con el auxilio de ChatGPT 4.0 fue sometido a validación rigurosa por dos revisores independientes, con experiencia en la conducción de revisiones de alcance y familiaridad con métodos de análisis cualitativo.

El proceso de categorización siguió los principios del análisis de contenido cualitativo básico, según lo recomendado en las directrices actualizadas del JBI para revisiones de alcance^11^, incluyendo las etapas de codificación abierta, desarrollo de categorías y revisión cruzada.

El *prompt* utilizado para auxiliar en la categorización fue el siguiente: “Actúa como un especialista en revisión de alcance. A continuación, se presentará una tabla con títulos y resúmenes de estudios incluidos. Con base en el contenido, identifica temas emergentes y agrupa los estudios en categorías temáticas, justificando cada agrupamiento con base en la similitud conceptual”.

Los datos no fueron enviados vía *upload* directo a la IA, sino que se organizaron en una tabla con la información de los estudios (autores, título, resumen y palabras clave), e ingresaron manualmente en el entorno seguro de la herramienta ChatGPT 4.0.

El contenido generado se trató como apoyo a la codificación inicial, y no como categorización final. La validación final de las categorías y subcategorías fue realizada por el equipo de investigadores, con registro de las decisiones en acta y planillas de trazabilidad. Este proceso buscó hacer la etapa de análisis más eficiente y transparente, sin comprometer el rigor metodológico. Las categorías derivadas se discutieron posteriormente en reuniones del equipo, garantizando el principio de triangulación de los datos. El uso de la IA fue, por lo tanto, complementario, asistido y supervisado, y no sustitutivo del análisis humano.

Paralelamente, se llevó a cabo un análisis cuantitativo simple, en el cual se calcularon frecuencias y porcentajes para describir la distribución de los datos en relación con las variables clave, como temas abordados y características demográficas de los estudios. Además, con el apoyo de la IA, fue más sencilla la elaboración de figuras, que se generaron a partir de CANVA, haciendo la visualización de los resultados más clara y accesible. Estas representaciones visuales facilitaron la interpretación de los resultados, proporcionando una visión amplia y clara de las evidencias recolectadas.

Los resultados del proceso de selección de los estudios se sintetizaron en un diagrama de flujo PRISMA-ScR^1^. 

### Resultados

Se identificaron un total de 602 registros en las bases *PubMed* (n=132), LILACS (n=62), CINAHL (n=55), Embase (n=150), Scopus (n=57), WOSCC (n=41), *Cochrane* (n=5) y *Google Scholar* (n=100). Tras la exportación a *EndNote*, se eliminaron 160 registros duplicados, lo que resultó en 442 estudios que pasaron al proceso de cribado en *Rayyan*. En la primera fase del cribado, realizada por título y resumen, se excluyeron 262 registros, lo que condujo 180 estudios a la fase de lectura en texto completo, de los cuales dos no pudieron recuperarse. En esta etapa, además se excluyeron 35 estudios tras la lectura completa. Así, 143 estudios fueron incluidos para la extracción y análisis de datos, como se ilustró en la Figura 1.

**Figura 1:** Diagrama de flujo de identificación, selección e inclusión de estudios de la revisión de alcance según las directrices del PRISMA-ScR^1^. Alfenas, MG, Brasil, 2024

La distribución de los estudios por año de publicación reveló tendencias significativas sobre la evolución de la producción científica relacionada con las revisiones de alcance. El mayor pico de publicaciones ocurrió en 2023 (41; 28,7%), seguido por 2021 (36; 25,2%) y 2022 (30; 21,0%). Hubo también una producción relevante en 2020 (14; 9,8%) y 2019 (9; 6,3%). Los años anteriores presentaron una menor cantidad de publicaciones, como en 2018 (4; 2,8%), 2017 (2; 1,4%), 2016 (1; 0,7%) y 2015 (3; 2,1%). Hasta la fecha de la búsqueda en las fuentes de información, tres (2,1%) estudios fueron identificados para el año 2024.

El análisis de los idiomas de publicación de los 143 estudios reveló una significativa diversidad lingüística en la producción científica. El idioma inglés se destacó como el más utilizado, con 38 (26,6%) estudios publicados exclusivamente en este idioma. La combinación de “inglés, portugués y español” apareció como la más frecuente, con 46 (32,2%) publicaciones. El uso combinado de “portugués e inglés” también fue expresivo, con 39 (27,3%) estudios. El portugués fue el idioma exclusivo de 18 (12,6%) estudios, mientras que el español fue exclusivo de apenas un estudio (0,7%), así como la combinación de “portugués y español”, que también apareció en una publicación (0,7%).

La Figura 2 ilustró los procedimientos metodológicos adoptados en revisiones de alcance realizadas por enfermeros en Brasil, destacando el uso de varias prácticas y tendencias importantes. La mayoría de los estudios (140; 97,9%) identificó claramente en sus métodos que se trataba de una revisión de alcance, y 129 (90,2%) hicieron esta identificación directamente en el título del estudio. El verbo “mapear” fue el más utilizado, apareciendo en 53 objetivos, lo que representó 37,1% del total. El marco metodológico más empleado fue el JBI, utilizado en 106 (74,1%) estudios, seguido por Arksey y O’Malley, con 10 (7,0%) estudios. Además, una combinación del JBI con las orientaciones de Arksey y O’Malley fue utilizada en cinco (3,5%) estudios. La mayoría de los estudios (87; 60,8%) hizo uso de PRISMA-ScR para describir sus investigaciones. Una proporción significativa de los estudios (92; 64,3%) adoptó un enfoque estructurado, con la definición clara de las etapas específicas de la revisión de alcance. Sin embargo, apenas 56 (39,2%) de los estudios mencionaron el registro del protocolo. Entre los estudios, 116 (81,1%) utilizaron el mnemónico PCC para estructurar sus revisiones de alcance. La pregunta de investigación fue mencionada en 137 (95,8%) estudios, mientras que apenas 30 (21,0%) incluyeron subpreguntas de investigación.

**Figura 2:** Panorama metodológico de las revisiones de alcance en la enfermería brasileña. Alfenas, MG, Brasil, 2024

La gran mayoría de los estudios (106; 74,1%) mencionó el uso de filtros y limitadores en sus estrategias de búsqueda. El filtro más utilizado fue la combinación de recorte temporal e idioma, aplicada en 32 (30,2%) estudios. Enseguida, el filtro por idioma aislado fue mencionado en 28 (26,4%) estudios. Se informaron otros filtros, incluyendo: recorte temporal aislado en 9 (8,5%) estudios, combinación de filtros por idioma y tipos de fuentes en 5 (4,7%) estudios, y filtro por documentos disponibles en texto completo combinado con idioma, presente en 6 (5,7%) estudios. Filtros menos frecuentes abarcaron la combinación de idioma, fuentes y documentos disponibles en texto completo en 1 (0,9%) estudio, además de combinaciones que involucraron recorte temporal y fuentes, o recorte temporal y documentos en texto completo, cada una mencionada en 1 (0,9%) estudio. El filtro que involucró solo tipos de fuentes fue utilizado en 3 (2,8%) estudios. Además, 6 (5,7%) estudios no detallaron las especificidades de los filtros utilizados.

Solo 14 (9,8%) estudios reportaron la realización de una prueba piloto de la estrategia de búsqueda. La estrategia de búsqueda fue desarrollada en tres etapas en 44 (30,8%) estudios. Más de la mitad de los estudios (85; 59,4%) utilizó la literatura gris como fuente de información, y la búsqueda manual en fuentes específicas fue mencionada en 26 (18,2%) estudios. La búsqueda por las referencias de los estudios incluidos fue empleada en 79 (55,2%) estudios. La estrategia de búsqueda fue presentada en la mayoría de los estudios (105; 73,4%). La selección de los estudios por título y resumen (Fase 1) y por lectura en texto completo (Fase 2) fue ampliamente practicada, con 115 (80,4%) y 116 (81,1%) de los estudios indicando esas fases, respectivamente. La selección por dos revisores fue mencionada en 105 (73,4%) de los estudios.

En cuanto a la distribución de los estudios seleccionados e incluidos en las revisiones de alcance, categorizándolos en cuatro intervalos distintos con base en el número de estudios, los intervalos bajos y moderados (1-50 estudios) dominaron la distribución de las revisiones de alcance, representando, en conjunto, 117 (81,9%) estudios. Las revisiones que involucraron un número mayor de estudios (51-100) fueron menos comunes, con 18 (12,5%) estudios, mientras que las revisiones extremas (más de 100 estudios) fueron bastante raras, sumando apenas 5,6%.

La Figura 3 abordó el proceso de extracción de datos. Primeramente, se observó que 57 (39,9%) estudios adoptaron el formulario del JBI, uno de los estándares más reconocidos para la extracción sistemática de datos. En relación con la inclusión de elementos del PCC en los formularios de extracción, 73 (51%) de los estudios mencionaron explícitamente su aplicación. Otro punto relevante fue la adhesión a los protocolos planificados. Solo 1 (0,7%) reportó desviación de protocolo en la extracción de datos. En lo que concierne al uso de tecnologías, apenas 17 (11,9%) estudios mencionaron explícitamente el uso de software para la extracción de datos, como *Microsoft Excel*, ATLAS.ti^®^, *web*QDA y *Apple Numbers*. En cuanto al número de investigadores involucrados en la extracción de datos, 70 (49%) estudios describieron esa información.

**Figura 3:** Ítems de los formularios y *software* utilizados en el proceso de extracción de datos de las revisiones de alcance en la enfermería brasileña. Alfenas, MG, Brasil, 2024

La Figura 4 ofreció una visión amplia sobre el proceso de análisis de datos, destacando tanto el software utilizado como los métodos de análisis empleados. Se observó que los principales programas empleados para el análisis de datos incluyeron herramientas cualitativas como ATLAS.ti^®^, MAXQDA, *web*QDA e IRaMuTeQ, además de *softwares* de uso más general, como *Microsoft Excel* y *Apple Numbers*. Estas herramientas desempeñaron un papel fundamental en la organización, codificación y análisis sistemático de los datos. En cuanto a los enfoques metodológicos adoptados, se verificó que el análisis descriptivo simple, así como el análisis temático y de contenido, se utilizaron ampliamente para organizar y categorizar los datos, respectivamente.

**Figura 4:** Proceso y *software* de análisis de datos de las revisiones de alcance en la enfermería brasileña. Alfenas, MG, Brasil, 2024

La Figura 5 destacó las principales formas de presentación de los resultados en revisiones de alcance en Brasil, con un predominio del uso de cuadros y categorías temáticas, que organizaron y sintetizaron información compleja, facilitando comparaciones e identificación de patrones. Tablas y gráficos también fueron ampliamente utilizados.

**Figura 5:** Formas de presentación de los resultados en revisiones de alcance en la enfermería brasileña. Alfenas, MG, Brasil, 2024

Las revisiones de alcance examinadas evidenciaron una expresiva diversidad de temas, agrupables en tres ejes principales: clínico-asistencial, poblacional y educativo-político. En el ámbito clínico, se destacaron producciones orientadas a cuidados paliativos, salud mental, seguridad del paciente, infecciones asociadas a la atención y procesos diagnósticos de enfermería.

En relación con enfoques emergentes, hubo un énfasis creciente en prácticas avanzadas de enfermería, tecnologías digitales aplicadas a la formación y a la asistencia, así como enfermería forense. En el plano poblacional, los estudios contemplaron grupos en situación de vulnerabilidad, como personas mayores, personas LGBTI+, pueblos indígenas, personas privadas de libertad, migrantes y refugiados. También se identificaron investigaciones centradas en condiciones clínicas específicas, como trastornos hipertensivos en la gestación, cáncer, enfermedad de Parkinson e infecciones por COVID-19 en múltiples perfiles, además de tópicos como adherencia terapéutica en la tuberculosis y estrategias de manejo del dolor.

En el dominio formativo y ético-político, se abordaron metodologías activas, simulación clínica, uso de juegos digitales, estilos de aprendizaje, derechos humanos en el cuidado a personas con trastornos mentales y acciones de preservación forense en contextos de violencia. Además, surgieron análisis relativos a la gestión y formulación de políticas públicas, como modelos de organización en salud mental, fiscalización profesional, sostenibilidad hospitalaria y acceso a servicios en regiones de frontera. Este panorama temático reafirma la relevancia de las revisiones de alcance en la consolidación del conocimiento en enfermería y en la orientación de prioridades para futuras agendas de investigación.

## Discusión

Este es el primer mapeo en cuantificar e interpretar la evolución metodológica de las revisiones de alcance producidas por enfermeros brasileños. El análisis temporal reveló un crecimiento expresivo de las revisiones de alcance publicadas por enfermeros brasileños en los últimos tres años (2021-2023). Tal expansión parece haber sido impulsada por respuestas a crisis globales, incluida la pandemia de COVID-19, así como por la necesidad de abordajes abarcadores para cuestiones de salud complejas, características inherentes a las revisiones de alcance^19^. Como ya lo sugiere la literatura, la flexibilidad de este enfoque en escenarios de conocimiento de rápida evolución^15^ viene consolidando la metodología en el campo de la enfermería nacional.

Sin embargo, el avance cuantitativo no fue acompañado por una mejora proporcional en la calidad metodológica. Aunque 90,2% de los estudios indicó en el título tratarse de revisión de alcance y 74,1% adoptó el marco JBI, solo 60,8% utilizó la guía PRISMA-ScR, y 39,2% mencionó el registro de protocolo. En casi dos quintos de las publicaciones, por lo tanto, persiste un riesgo relevante de baja transparencia y reproducibilidad, escenario igualmente descrito en revistas internacionales de Enfermería^13^.

Estos hallazgos revelan oportunidades de perfeccionamiento en la formación de investigadores, sobre todo en cuanto a la importancia de la adhesión rigurosa a las directrices metodológicas, con miras a ampliar la robustez y la transparencia de las revisiones y a maximizar su impacto en la práctica clínica, educativa y de gestión^1^ ^,^ ^10^ ^,^ ^12^.

Aunque la mayoría de los estudios describió las etapas de la revisión de alcance, una proporción significativa omitió detalles relevantes del proceso, lo que evidenció la necesidad de mayor claridad y sistematización^8^ ^,^ ^13^. El empleo del mnemónico PCC en más de la mitad de las revisiones reforzó su utilidad en la formulación de la pregunta, facilitando la identificación de elementos esenciales y la aplicabilidad de los hallazgos^9^. Cabe destacar que la incorporación del PCC contribuye a la elaboración de un objetivo más claro y orientado al objeto de estudio^9^ ^,^ ^20^.

La aplicación de filtros y limitadores en las búsquedas fue una práctica común. Sin embargo, pocos autores justificaron esas elecciones. Este silencio metodológico puede introducir sesgo y limitar la reproducibilidad, especialmente cuando se restringen idiomas o fechas sin explicitar el motivo^5^ ^,^ ^21^. Para el contexto brasileño, marcado por diversidad cultural y lingüística, justificar filtros de idioma y tiempo es fundamental para evitar la exclusión de evidencias regionales relevantes.

La estrategia de búsqueda en tres etapas, conforme a la recomendación del JBI^9^, y la revisión por pares de las estrategias de búsqueda, preconizada por la directriz PRESS^22^, fueron poco referidas, lo que puede señalar limitaciones operativas o de formación de los investigadores^5^ ^,^ ^8^ ^,^ ^22^. Prácticas complementarias como la búsqueda manual y el rastreo de referencias también fueron subutilizadas, lo que puede haber reducido la amplitud de los estudios^23^.

La mayoría de las revisiones de alcance brasileñas analizó entre 21 y 50 estudios, lo que señaló un abordaje suficiente para la robustez analítica sin sacrificar la profundidad de los datos^22^. Síntesis más concisas o muy extensas fueron menos frecuentes, lo que sugiere preferencia por alcances focalizados y gestionables^23^.

En cuanto a la extracción y al análisis de datos, los autores emplearon estrategias diversas, con predominio de formularios adaptados del JBI. Solo una pequeña porción reportó el uso de tecnologías de apoyo, como software de análisis cualitativo u hojas de cálculo automatizadas, lo que revela la oportunidad de ampliar la adopción de recursos digitales^11^ ^,^ ^14^ ^,^ ^24^ ^-^ ^26^. El mismo patrón se observó en la presentación de los resultados, realizada mayoritariamente por medio de cuadros, gráficos y categorías temáticas. El empleo de recursos más interactivos, como *dashboards* o visualizaciones dinámicas, aún fue incipiente, lo que limita el alcance comunicativo de los mapeos.

En síntesis, aunque los investigadores han avanzado en la incorporación de directrices metodológicas, persiste una brecha entre rigor científico e innovación tecnológica. La predominancia de procesos manuales, la baja tasa de registros de protocolo y la subutilización de *software* especializado evidencian desafíos que deben superarse para elevar la consistencia y la calidad de las revisiones de alcance^25^.

Este estudio presentó limitaciones relacionadas con la restricción de las bases de datos consultadas, lo que pudo haber reducido la amplitud de los resultados, y con la exclusión de revisiones realizadas en otros contextos disciplinares o internacionales, restringiendo la generalización de los hallazgos. A pesar de esos límites, ofreció contribuciones relevantes para la práctica y la investigación en Enfermería en Brasil, al promover la estandarización metodológica y al incentivar la adopción de tecnologías que fortalezcan la práctica basada en evidencias.

Al trazar un panorama actualizado del desarrollo de las revisiones de alcance conducidas por enfermeros brasileños, este estudio enfatizó la importancia de la formación continua, con foco en la cualificación metodológica y la integración de tecnologías^10^. La diseminación sistemática de guías actualizadas (JBI y PRISMA-ScR), aliada a iniciativas de innovación, como el uso de *software* de análisis cualitativo e IA para cribado, tiene potencial para fortalecer la producción científica en Enfermería y ampliar la utilidad de esas revisiones en la práctica clínica, educativa y de gestión.

La adopción experimental de inteligencia artificial en la etapa de categorización, realizada en este trabajo, demostró viabilidad y eficiencia, constituyendo un camino promisorio para cualificar futuras revisiones de alcance conducidas por enfermeros brasileños. Conforme a la orientación metodológica recién publicada por el grupo JBI, que describe cuatro niveles progresivos de automatización y recomienda registrar en el protocolo las herramientas elegidas, con piloto y capacitación previos, la incorporación de IA debe ir acompañada de validación formal y reporte transparente para garantizar trazabilidad y seguridad metodológica^24^. Investigaciones subsecuentes deberían evaluar el impacto de esta tecnología sobre el tiempo de ejecución, la exhaustividad y la claridad de los resultados, así como explorar su aplicabilidad en diferentes áreas de la Enfermería.

## Conclusión

Este estudio evidenció que, en las revisiones de alcance conducidas por enfermeros brasileños, los datos fueron mayoritariamente extraídos por medio de formularios adaptados del JBI, con bajo uso de *software* de apoyo; el análisis se concentró en síntesis descriptivas y categorización temática; y la presentación de los resultados privilegió cuadros y tablas, recurriendo rara vez a visualizaciones interactivas.

Este panorama refuerza la importancia de directrices estandarizadas (JBI, PRISMA-ScR) y de tecnologías avanzadas, incluida la IA para cribado y *dashboards* para diseminación, a fin de elevar la transparencia, la replicabilidad y el impacto clínico de las revisiones de alcance. Las inversiones en formación continua deberán centrarse no solo en la adhesión a guías metodológicas, sino también en la capacitación para herramientas digitales que optimicen la extracción, el análisis y la comunicación de los hallazgos.

Futuras investigaciones deberán evaluar (i) el efecto del uso de IA en la rapidez y la exhaustividad de la extracción de datos, (ii) la contribución del *software* cualitativo a la profundidad analítica y (iii) el valor de las visualizaciones interactivas para la toma de decisiones en escenarios de práctica y gestión en la Enfermería brasileña.

## Declaración de Disponibilidad de Datos:

Todos los datos generados o analizados durante este estudio están incluidos en este artículo publicado.
